# Feedforward prediction error signals during episodic memory retrieval

**DOI:** 10.1038/s41467-020-19828-0

**Published:** 2020-11-27

**Authors:** Rafi U. Haque, Sara K. Inati, Allan I. Levey, Kareem A. Zaghloul

**Affiliations:** 1 Surgical Neurology Branch, NINDS, National Institutes of Health, Bethesda, MD 20892 USA; 2 Office of the Clinical Director, NINDS, National Institutes of Health, Bethesda, MD 20892 USA; 3 Department of Neurology, Emory University, Atlanta, GA 30322 USA

**Keywords:** Cortex, Long-term memory

## Abstract

Our memories enable us to form expectations for our future experiences, yet the precise neural mechanisms underlying how we compare any experience to our memory remain unknown. Here, using intracranial EEG recordings, we show that episodic memories formed after a single visual experience establish expectations for future experience within neocortical-medial temporal lobe circuits. When subsequent experiences violate these expectations, we find a 80–120 Hz prediction error signal that emerges in both visual association areas and the medial temporal lobe. Critically, this error signal emerges in visual association areas first and then propagates to the medial temporal lobe. This error signal is accompanied by alpha coherence between the two regions. Our data therefore suggest that internal models formed from episodic memories are generated throughout the visual hierarchy after just a single exposure, and that these internal models are then used for comparison with future experiences.

## Abstract (web summary)

Haque et al. demonstrate that the episodic memory of a single visual scene is sufficient for humans to recognize if a visual scene has subsequently changed. A prediction error signal first arises in the visual association cortex when individuals recognize these changes.

## Introduction

When exposed to any experience, we rely upon our memories to set our expectations. These expectations determine the extent to which any experience involves new information. For example, when we enter our home, we rarely consider the fact that the couch is still next to the wall since that arrangement has been embedded in our memory. Conversely, coming home to find the couch on the other side of the room would be surprising and violate our expectations for how the room should look based on our memory.

From a computational perspective, it would be efficient to dedicate more cognitive resources when an experience is novel or if it violates our expectations^1–4^. This is because identical experiences contain no new information whereas novel or unexpected experiences do. This hypothesis, termed predictive coding, has been articulated in computational and theoretical accounts of brain processing and posits that neural activity is optimized to maximize information^2,5–7^. A key requirement of this hypothesis is that the expectations and predictions to which new experiences are compared are stored in memory. For simple visual and auditory stimuli, these expectations are learned through a lifetime of observing and processing the statistical regularities of the natural world and are stored as memory in the brain networks and synaptic weights of primary visual and auditory cortex^8^. Efficiently processing new sensory inputs involves comparing those inputs to our expectations for how the visual and auditory world should appear^2,5–7,9^.

While the principles of predictive coding have been supported and described in empirical accounts of lower level sensory processing, it is not clear if and how similar mechanisms may be engaged when considering single episodes or events that we experience. We rely upon episodic memory to encode and remember these events^10,11^. An important distinction between episodic memory and memory of the statistics of the natural world is that we can form episodic memories even when the experiences we are remembering involve just a single exposure^10^. These memories are generated and stored through feedforward and feedback interactions between the neocortex and medial temporal lobe (MTL)^12–14^. When presented with a similar experience, the previous experience can be retrieved through autoassociative reactivation of these neocortical–MTL representations^12–16^. This framework therefore provides an internal memory to which new experiences may be compared. Any difference between past and present experience should violate the expectations set by our episodic memory and therefore signal an error in the predictions we had established for the new experience.

Here we examine whether comparing a new experience to an episodic memory indeed evokes a prediction error signal, and the neural mechanisms that underlie this process in the human brain. We were specifically interested in how such error signals are represented in the neocortex and the MTL, as the interactions between them underlie our ability to encode and retrieve episodic memories^12,13^. We presented participants with images of natural scenes and objects that they encoded into memory. We then tested their memory for these images by presenting them with the same images. Critically, during testing, we manipulated some of the images to either remove items from or add items to the original scene. Successfully recognizing this manipulation therefore requires participants to not only retrieve the past visual experience but to also then compare the retrieved memory with the present image. We examined changes in intracranial electroencephalography (iEEG), captured through subdural electrodes implanted for seizure monitoring, and how these changes were temporally related to eye movements that participants made as they scanned the new scenes during recognition testing. Recognizing manipulated images, and therefore successfully identifying a difference between past and present experience, evoked a high frequency band prediction error signal in visual association cortex that then propagated toward the MTL. During successful recognition of these manipulations, this error signal was also accompanied by elevated low frequency coherence between the neocortex and MTL. Our results therefore provide a direct account of how violations of the expectations set by the episodic memory of a previous experience are encoded in the human brain.

## Results

**Fig. 1:**
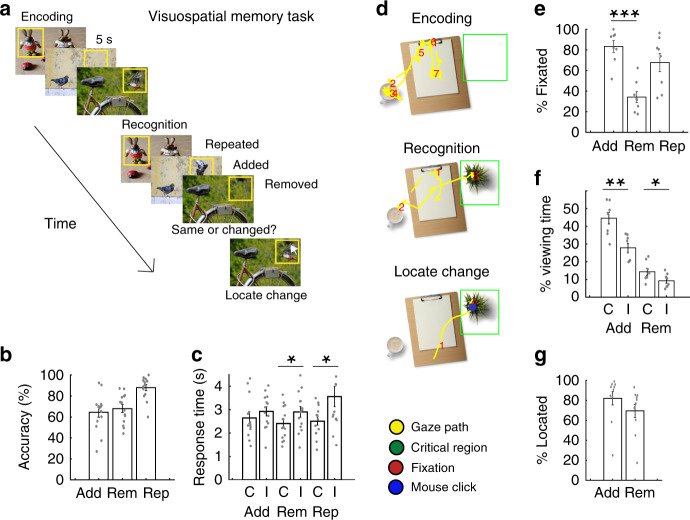
Visuospatial memory task. **a** Participants viewed and encoded into memory a series of images. During recognition testing, participants indicated whether the images were manipulated (items added or removed) or whether they were identical to the encoded images. Participants subsequently identified the location of the manipulation using a mouse click. **b** Probability of successful recognition for the added (Add), removed (Rem), and repeated (Rep) conditions across participants. **c** Mean response times for correct (C) and incorrect (I) responses for added, removed, and repeated conditions across 14 participants. Response times were significantly faster when participants correctly recognized removed and repeated images compared to when they were incorrect (removed, *t*(13) = −2.31, *p* = 0.038, repeated *t*(12) = −2.85, *p* = 0.014). **d** Representative eye-movements during encoding, recognition, and identification of the location of the manipulation. We converted the gaze path (yellow) into fixations (red) and calculated the percentage of fixations within the critical region of each image (green box). **e** Probability of fixation within the critical region for correctly recognized added, removed, and repeated items across 8 participants. We selected a random item as the critical region for the repeated condition to assess the probability of baseline fixations. Participants spent at least one fixation on a greater percentage of the successfully recognized added compared to removed items (*t*(7) = 11.2, *p* = 10^−5^). **f** Probability of viewing time within critical region for correctly and incorrectly identified images across 8 participants. Participants spent a greater percentage of time viewing both the added item and the region of the removed item during correct compared to incorrect trials (added, *t*(7) = 4.93, *p* = 0.002; removed, *t*(7) = 2.52, *p* = 0.040). **g** Probability of mouse click within critical region for correctly recognized added and removed conditions across participants. All error bars indicate standard error of mean. Asterisks (*, **, ***) indicate significance at *p* < 0.05, *p* < 0.01, and *p* < 0.001, respectively; two-sided paired *t* tests.

Fourteen participants (7 males, 40.9 ± 12 years) with intracranial electrodes placed for seizure monitoring performed a visuospatial recognition memory task (Fig. 1a; see “Methods”). During the encoding portion of the task, we presented images of natural scenes containing different items to participants and instructed them to remember the images. We then subsequently presented the same images during the recognition phase of the task and tested their memory for the images. We either added or removed an item from some of the images that were presented during the recognition phase and instructed the participants to indicate whether each image was identical to the one they had encoded or if it had been manipulated. We therefore designated the three different types of images presented during recognition testing as repeated, added, or removed versions of the original images based on the manipulation we performed. If the participant indicated that the image had been manipulated, we then instructed them to identify the location of the manipulation using a mouse click on the screen (Fig. 1a).

Participants successfully recognized 65 ± 5, 68 ± 4, and 88 ± 3% of the added, removed, and repeated images, respectively, during testing with a mean response time of 2.65 ± 0.23, 2.41 ± 0.19, and 2.51 ± 0.17s (Fig. 1b, c). Response times were significantly faster when participants correctly recognized removed and repeated images compared to when they were incorrect (removed, *t*(13) = −2.31, *p* = 0.038, paired *t* test; repeated *t*(12) = −2.85, *p* = 0.014) but not when comparing correct and incorrect added images (*t*(13) = −1.36, *p* = 0.195).

In a subset of participants (*n* = 8), we recorded the location of each participant’s gaze as they scanned the images during recognition testing (Fig. 1d; see “Methods”). Participants spent at least one fixation on 83 ± 4% of successfully recognized added items, but on only 34 ± 4% of the regions in which items were removed when successfully recognizing a removed image (*t*(7) = 11.2, *p* = 10^−5^; Fig. 1e). However, participants spent a greater percentage of time viewing both the added item and the region of the removed item during correct compared to incorrect trials (added, *t*(7) = 4.93, *p* = 0.002; removed, *t*(7) = 2.52, *p* = 0.040, paired *t* test; Fig. 1f). In addition, participants were able to correctly identify the location of the manipulation when successfully recognizing the items had been added or removed in 82 ± 6 and 70 ± 6% of the trials, respectively (Fig. 1g). These data together suggest that participants were able to successfully recognize when and where an image was manipulated even though they were more likely to explicitly fixate on the manipulation only when an item was added.

### 80–120 Hz power progresses down the visual hierarchy and reflects specific visual experience

**Fig. 2:**
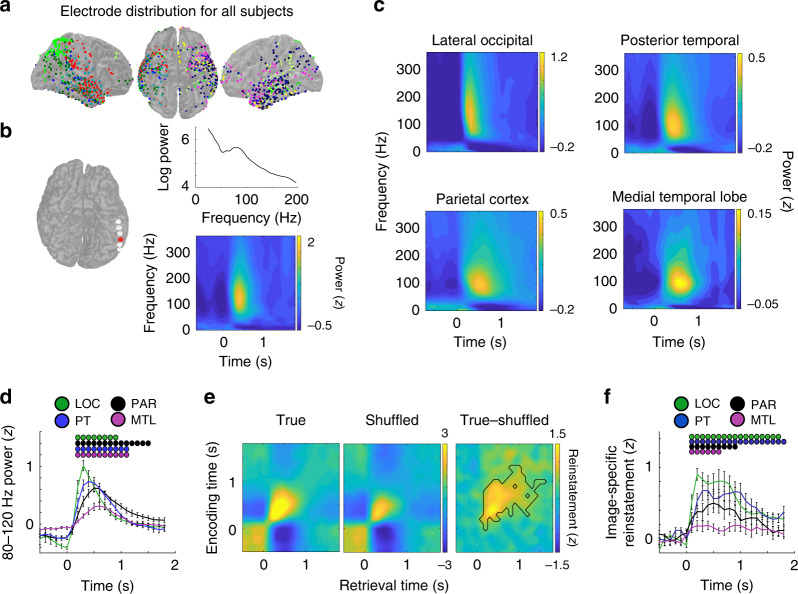
Feedforward transmission of 80–120 Hz power between cortex and medial temporal lobe reflect specific visual experiences. **a** Intracranial electrode locations for all 14 participants. Each color corresponds to an individual participant. **b** In a representative electrode in the posterior temporal lobe (red), a single trial exhibits an increase in power in the 80–120 Hz band during recognition testing (top). Across all trials in this electrode, images during the recognition period elicited increases in 80–120 Hz power. **c** Across participants, image presentation during the recognition period evoked increases in 80–120 Hz power in the lateral occipital cortex (LOC), posterior temporal lobe (PT), parietal cortex (PAR), and medial temporal lobe (MTL). **d** Average time course of 80–120 Hz power during the recognition period in each brain region across participants (200 ms sliding windows, 50% overlap; image appears at *t* = 0). Dots indicate significant increases in 80–120 Hz power compared to baseline, corrected for multiple comparisons across 6 LOC, 8 PAR, 10 PTL, and 10 MTL participants (*p* < 0.05, permutation procedure). **e** Reinstatement of 80–120 Hz power distributed across all electrodes, averaged across all participants. The difference in average reinstatement between the true and shuffled distributions reflects image-specific reinstatement across all participants (black outline, *p* < 0.05, permutation procedure). **f** Time series of average image-specific reinstatement across 6 LOC, 8 PAR, 8 PT, and 10 MTL participants during the recognition period. Dots indicate significant increases in image-specific reinstatement corrected for multiple comparisons (*p* < 0.05, permutation procedure; image appears at *t* = 0). All error bars indicate standard error of mean.

We examined iEEG recordings captured from intracranial electrode contacts in all participants as they performed the visuospatial recognition task (Fig. 2a; see “Methods”). In a representative example electrode in the posterior temporal cortex (PT), we found an increase in high frequency power that was centered at 80–120 Hz and that was reliably detected across all trials during the recognition period (Fig. 2b). We divided electrode contacts in each participant into four regions of interest—lateral occipital cortex (LOC), parietal cortex (PAR), PT, and the MTL—based on the known feedforward organization within the visual hierarchy^17^ (Supplementary Fig. 1). In each of these regions across participants, we observed a consistent increase in high frequency power centered around 80–120 Hz when averaged across all trials during the recognition period (Fig. 2c). This frequency band has been previously implicated in human memory retrieval^16^, and given the patterns of spectral power here, we focused our subsequent analyses on changes in spectral power within this band of high frequency activity.

When examining all recognition trials, we found that 80–120 Hz power exhibited a significant increase above baseline 200 ms after image presentation in all regions (*p* < 0.001, permutation procedure; see “Methods”; Fig. 2d). This rise in 80–120 Hz power peaked within 600 ms in all regions but peaked at a significantly earlier time following image presentation in LOC and PT compared to MTL (*p* < 0.05, permutation procedure). The rise in 80–120 Hz activity was significantly faster in LOC, PAR, and PT compared to MTL (LOC vs MTL, *t*(14) = 2.26, *p* = 0.04; PAR vs MTL, *t*(16) = 3.14, *p* = 0.006; PT vs MTL *t*(17) = 2.84, *p* = 0.011; see “Methods”), suggesting that image presentation during the recognition period evokes a rise in 80–120 Hz power that progresses down the visual hierarchy.

Given that the images presented during recognition testing were similar, but not identical, to the images presented during encoding, we investigated whether the patterns of 80–120 Hz power that arose along the visual hierarchy following image presentation during recognition testing were also similar to the patterns of 80–120 Hz power present during encoding. For each image presented during encoding and again during retrieval, we computed the distributed pattern of 80–120 Hz power across all electrode contacts at each time point following image presentation. We computed how similar the distributed power at each time point during recognition testing was to the power present at every time point when encoding the same image (see “Methods”). We found that viewing the same image, regardless of whether the image was manipulated, reinstated the distributed pattern of 80–120 Hz power that was present during encoding across participants (Fig. 2e).

We compared the true reinstatement of these distributed patterns of 80–120 Hz power to the reinstatement observed after shuffling the trial labels in order to assess whether this reinstatement was specific to each individual image (see “Methods”). Viewing the images during recognition testing significantly reinstated the specific distributed patterns of activity for each image as compared to the shuffled trials beginning 100 ms after the image presentation (*p* < 0.001, permutation test; Fig. 2e). We found image-specific reinstatement even when considering each individual region of interest separately (*p* < 0.05, permutation test; Supplementary Fig. 2a). We then computed a time series of the mean level of reinstatement during recognition testing within each region by using all encoding epochs that demonstrated image-specific reinstatement when considering all electrodes. Across participants, we found that viewing the image during recognition testing resulted in significant image-specific reinstatement in the LOC, PT, PAR, and MTL (*p* < 0.05, permutation test; Fig. 2f), suggesting that the progression of high frequency power down the visual hierarchy contains information regarding the specific image being viewed. We did not find any differences in reinstatement between the conditions during the first 500 ms when reinstatement was maximal (Supplementary Fig. 2).

### 80–120 Hz power increases when the present visual experience differs from the remembered experience

**Fig. 3:**
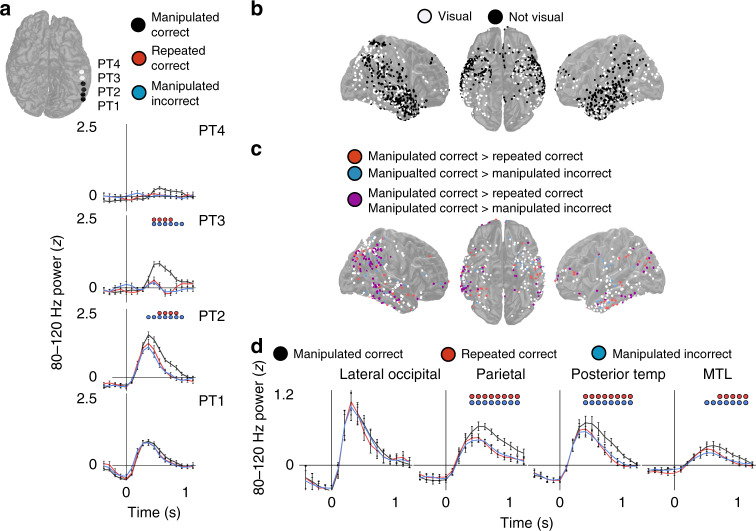
Increases in 80–120 Hz power in visual association cortex and MTL reflect differences between present and remembered visual experience. **a** Time series of 80–120 Hz power for a set of posterior temporal electrodes in an example participant during 142 manipulated correct (black), 122 manipulated incorrect (blue), and 82 repeated correct (red) recognition trials (200 ms sliding windows, 50% overlap; image appears at *t* = 0). Blue and red dots indicate significant differences in 80–120 Hz power between manipulated correct and manipulated incorrect and repeated correct condition, respectively (*p* < 0.05, permutation procedure). **b** All electrodes across all participants demonstrating significant increases in 80–120 Hz power from baseline (white; *p* < 0.05, permutation procedure corrected for multiple comparison within each electrode). **c** All visually responsive electrodes in each participant (white) demonstrating significant increases in 80–120 Hz power for the manipulated correct compared to the manipulated incorrect (blue), manipulated correct compared to the repeated correct (red), or both (purple; *p* < 0.05, permutation procedure corrected for multiple comparison within each electrode). **d** Time courses of 80–120 Hz power for the manipulated correct, manipulated incorrect, and repeated correct conditions across 6 LOC, 8 PAR, 10 PT, and 10 MTL participants during the recognition period (200 ms sliding windows, 50% overlap; image appears at *t* = 0). Blue and red dots indicate significant differences in 80–120 Hz power between manipulated correct and manipulated incorrect and repeated correct condition, respectively (*p* < 0.05, permutation procedure). All error bars indicate standard error of mean.

Participants in our task were able to correctly identify when a visual image was manipulated compared to the image they had remembered. We were interested in examining the neural mechanisms underlying this ability to recognize the difference between past and present visual experience. We therefore compared the image-specific increases in 80–120 Hz power that progress down the visual hierarchy between conditions. In an example participant, we examined a set of electrodes arranged in linear strip from posterior to anterior regions of the PT. Viewing the manipulated images resulted in a significantly greater and more prolonged increase in 80–120 Hz power in individual electrode contacts when compared to viewing a repeated image or viewing an image that had been manipulated but was incorrectly identified as being repeated (*p* < 0.001, permutation test; see “Methods”; Fig. 3a). This difference was specific to two electrode contacts and not present on the most posterior contact and was more attenuated in the anterior contact, demonstrating that correctly identifying a difference between a presented and remembered image results in difference in 80–120 Hz power only within specific regions of the visual association cortex.

We examined the changes in 80–120 Hz power between conditions in all electrode contacts in all participants. We first identified any electrode contact that demonstrated a significant difference in 80–120 Hz power at any point during recognition testing compared to baseline when averaged across all trials (Fig. 3b). We found that 43% of electrode contacts across participants exhibited a significant increase in 80–120 Hz power from baseline (*p* < 0.05, permutation test; see “Methods”; 17% of electrode contacts showed a significant decrease). We designated these electrode contacts as visually responsive. Visually responsive electrode contacts were primarily located in LOC, PAR, PT, and MTL and were relatively absent from the anterior lateral temporal cortex. We then investigated how activity within these visually responsive electrodes changed between conditions. We found that 35 and 31% of visually responsive electrode contacts demonstrated significantly greater 80–120 Hz power at some point when viewing manipulated images that were correctly identified compared to repeated images and compared to images that were not correctly identified as manipulated, respectively (*p* < 0.05, permutation test; Fig. 3c, red and blue electrodes, respectively). In all, 21% of the electrodes exhibited a significant increase in both comparisons (*p* < 0.05, permutation test; Fig. 3c, purple electrodes). In contrast, <5% of electrode contacts demonstrated a significant decrease in 80–120 Hz power in any of these comparisons (*p* < 0.05, permutation test; Supplementary Fig. 3).

Although these data suggest that a large subset of electrode exhibit significantly greater 80–120 Hz power at some time point when viewing manipulated images, we were specifically interested in understanding the time course of these changes. We therefore examined the average time series of 80–120 Hz power in each condition across all visually responsive electrode contacts in each region. Across participants, as in the example set of electrodes, we found that electrodes within PT, PAR, and MTL exhibited significantly higher and more prolonged 80–120 Hz power when viewing and correctly identifying manipulated images than when viewing repeated or incorrectly identified manipulated images (*p* < 0.01, permutation test; Fig. 3d). The differences observed in the MTL were specifically localized to the parahippocampal gyrus and entorhinal cortex, but not the hippocampus (*p* < 0.01, permutation test; Supplementary Fig. 4). We did not observe a significant difference between conditions in the LOC (*p* > 0.05, permutation test). The differences observed between the conditions were specific to the 80–120 Hz frequency band (Supplementary Fig. 5), were not related to differences in trial counts (Supplementary Fig. 6), and reflected temporally discrete increases in 80–120 Hz power within each trial (Supplementary Fig. 7). Moreover, these differences were not present during the encoding period (Supplementary Fig. 8), and overall 80–120 Hz power was enhanced when viewing the images during recognition testing compared to encoding in PT, PAR, and MTL in all three conditions (Supplementary Fig. 9).

**Fig. 4:**
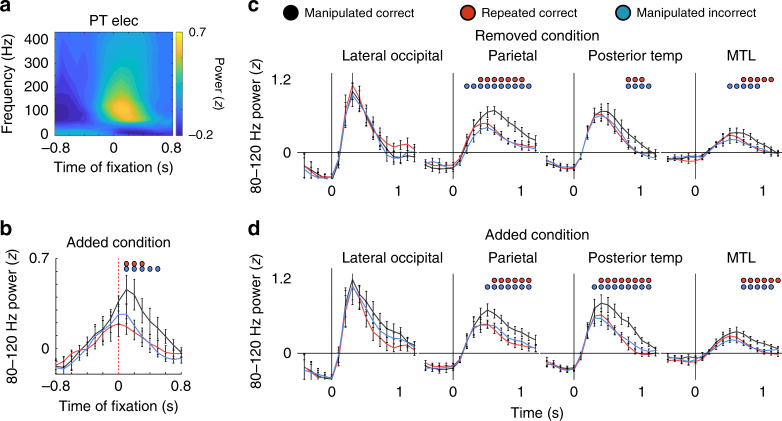
Increases in 80–120 Hz power are locked to eye fixations and emerge whether an item is added or removed. **a** Average spectrogram relative to the first fixation on successfully recognized added items for a representative electrode in PT. **b** Time courses of 80–120 Hz for visually responsive electrodes across participants for the added correct, added incorrect, and repeated correct conditions locked to the first fixation on the added item (200 ms sliding windows, 50% overlap; eye fixation at *t* = 0). Blue and red dots indicate significant differences in 80–120 Hz power between added correct and added incorrect and repeated correct condition across 8 participants, respectively (*p* < 0.05, permutation procedure). **c** Time courses of 80–120 Hz power for the removed correct, removed incorrect, and repeated conditions across 6 LOC, 8 PAR, 10 PT, and 10 MTL participants during the recognition period (200 ms sliding windows, 50% overlap; image appears at *t* = 0). Blue and red dots indicate significant differences in 80–120 Hz power between removed correct and removed incorrect and repeated correct condition, respectively (*p* < 0.05, permutation procedure). **d** Time courses of 80–120 Hz power for the added correct, added incorrect, and repeated conditions across 6 LOC, 8 PAR, 10 PT, and 10 MTL participants during the recognition period (200 ms sliding windows, 50% overlap; image appears at *t* = 0). Blue and red dots indicate significant differences in 80–120 Hz power between added correct and added incorrect and repeated correct condition, respectively (*p* < 0.05, permutation procedure). All error bars indicate standard error of mean.

In a subset of participants, we recorded eye movements during recognition testing in order to determine whether the observed changes in 80–120 Hz power were temporally related to viewing the manipulated item. We focused on the added condition because participants were significantly more likely to make a fixation to the added item than to the location of the removed item in manipulated images (Fig. 1e). In a representative example, we found that the increase in 80–120 Hz power locked to the time of the fixation on the manipulated item across all trials (Fig. 4a). Across participants, we found that fixating on the added item resulted in a significantly higher level of 80–120 Hz power immediately following the fixation during trials that were correctly identified as manipulated compared to incorrect trials and to trials with no manipulation (*p* < 0.001, permutation test; Fig. 4b and Supplementary Fig. 10). Hence, the observed differences in 80–120 Hz power appear to be triggered by viewing an item that was not present in a remembered visual image.

One concern regarding the differences we observed in 80–120 Hz power between manipulated and repeated images is that these differences could have been driven by the stimulus properties of the image presented during recognition testing. For example, some of the manipulated images contained items that were added to the original image, and the increases in 80–120 Hz power may simply be due to the additional visual input from the added item. To examine this possibility, we separately analyzed the trials in which manipulated images contained an added or removed item and compared them to the repeated images and to the corresponding incorrectly identified manipulated images. Both the added and removed conditions demonstrated similar significant increases in 80–120 Hz power compared to the repeated correct and manipulated incorrect conditions (*p* < 0.05, permutation test; Fig. 4c, d). These data therefore confirm that the observed increases in 80–120 Hz power arise due to manipulation of the image and are not related to specifically how the image had changed.

### Differences in 80–120 Hz power during manipulated images progress down the visual hierarchy

We next examined the time course of this 80–120 Hz difference signal that arises when viewing a manipulated image across brain regions. Our goal was to distinguish whether this signal progresses from posterior to anterior brain regions similar to routine visual processing^17^ or whether this difference signal is first detected in higher-order brain regions such as the MTL that are explicitly involved in encoding and retrieving the associations present in each image^13^.

**Fig. 5:**
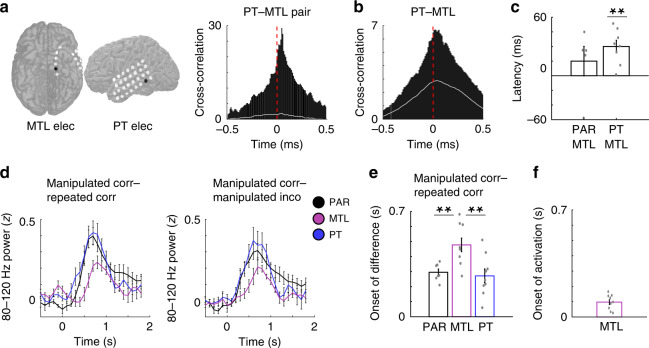
Differences in 80–120 Hz power between present and remembered visual experience emerge in visual association cortex earlier than in the MTL. **a** Representative cross-correlation of 80–120 Hz power between a PT and MTL electrode in an example participant. The chance cross-correlation for this electrode pair is indicated by the white line. **b** Average cross-correlation of 80–120 Hz power between the PT and MTL electrodes across participants. The chance cross-correlation for the average across all electrode pairs is indicated by the white line. **c** Average peak times (latency) of 80–120 Hz cross-correlograms for PT–MTL and PAR–MTL electrode pairs across participants. Average latency between PT and MTL electrodes across 8 participants was significantly greater than zero (*t*(7) = 4.78, *p* = 0.002, two-tailed paired *t* test). **d** Average time series of differences between the manipulated and repeated correct conditions (left) and manipulated correct and incorrect conditions (right) across 9 PT, 7 PAR, and 9 MTL participants (200 ms sliding windows, 50% overlap; image appears at *t* = 0). **e** Average estimated onset of differences in 80–120 Hz power between manipulated correct and repeated conditions in 9 PT, 7 PAR, and 9 MTL participants (left). The differences in 80–120 Hz power between manipulated correct and repeated conditions arose significantly earlier in PT and PAR compared to MTL (PT vs MTL, *t*(16) = −3.21, *p* = 0.005; PAR vs MTL, *t*(14) = −3.32, *p* = 0.005). Asterisks (**) indicate significance at *p* < 0.01; two-sided unpaired *t* test. **f** Average estimated onset of 80–120 Hz MTL power (activation) across 9 participants. All error bars indicate standard error of mean.

We first examined the direction of propagation of overall 80–120 Hz power between neocortical association areas and the MTL. In an example pair of electrodes located in the PT and MTL, we computed a cross-correlation of 80–120 Hz power using the first second of data from all trials (irrespective of condition) following image presentation during recognition testing (Fig. 5a). Across all trials in this example pair, the cross-correlation demonstrated a clear peak that was significantly greater than chance (*p* < 0.01, permutation procedure; see “Methods”) and that 80–120 Hz power in PT preceded MTL with a consistent delay. We repeated these cross-correlations across all participants using all visually responsive electrode pairs between brain regions. Across participants, we found a significant peak in the cross-correlation of 80–120 Hz power between PT and MTL (*t*(7) = 6.13, *p* = 0.0005, paired *t* test; see “Methods”; Fig. 5b) and between PAR and MTL (*t*(4) = 4.86, *p* = 0.008). We used the time of the peak of each cross-correlation to quantify the delay in 80–120 Hz activity between brain regions and averaged the peak delay across participants (Fig. 5c; see “Methods”). The increases in 80–120 Hz power in PT significantly preceded the increases in MTL by an average delay of 40 ± 8 ms across participants (*t*(7) = 4.78, *p* = 0.002, paired *t* test). We observed this delay between these two brain regions even when separating the retrieval trials by condition (51 ± 12, 32 ± 16, and 37 ± 18 ms for manipulated correct, repeated correct, and manipulated incorrect trials, respectively; *p* < 0.01, paired *t* test for each condition). No differences in delay were observed between the PAR and MTL (*t*(4) = 0.99, *p* > 0.05, paired *t* test).

We next examined whether the difference signals that arose in 80–120 Hz power when viewing an image that had been manipulated also progressed in a feedforward direction along the visual hierarchy. We visualized the time course of these differences in each brain region across participants and identified the first time point when the difference in 80–120 Hz power between manipulated correct and repeated conditions and between manipulated correct and incorrect trials deviated from zero (Fig. 5d; see “Methods”). Across participants, the differences in 80–120 Hz power between manipulated correct and repeated conditions arose significantly earlier in PT and PAR compared to MTL (PT vs MTL, *t*(16) = −3.21, *p* = 0.005, unpaired *t* test; PAR vs MTL, *t*(14) = −3.32, *p* = 0.005; Fig. 5e). These differences began at 271 ± 46 and 295 ± 21 ms after the image presentation in PT and PAR, respectively, but only started at 477 ± 45 ms in the MTL. We found the differences in 80–120 Hz power between manipulated and repeated conditions also reached 50% of the peak significantly earlier in PT and PAR compared to MTL (PT vs MTL, *t*(16) = −2.63, *p* = 0.018, unpaired *t* test; PAR vs MTL, *t*(14) = −2.81, *p* = 0.013). We also identified the time point that differences became significant for each electrode and compared these time points between regions across participants. We found the differences in PT became significant earlier than the MTL (*t*(16) = −2.45, *p* = 0.026). We found similar temporal patterns of activation when comparing manipulated correct and incorrect conditions (Supplementary Fig. 11), together demonstrating that the differences that are detected between past and present visual experience are captured by increases in 80–120 Hz power that also propagate from posterior to anterior brain regions along the visual hierarchy.

Although specific differences in activity between manipulated and repeated conditions arose in the MTL only after those differences were detected in the visual association cortex, we hypothesized that the ability to detect any difference between past and present experience required initially retrieving the past experience and therefore activation of the MTL. We therefore examined when the differences in 80–120 Hz power that we observed in PT and PAR occurred with respect to overall activation of the MTL. Based on the increase in 80–120 Hz power observed across all trials following the presentation of the image during recognition testing (Fig. 2d), we estimated the first time point at which MTL activity exceeded baseline as above (97.2 ± 16 ms; see “Methods”; (Fig. 5e). We then compared this time point to the first time points exhibiting a difference in activity between conditions in PT and PAR. In both cases, we found that overall activation of the MTL preceded the detection of a difference between past and present visual experience in the visual association cortex (PT vs MTL, *t*(16) = −3.58, *p* = 0.003; PAR vs MTL, *t*(14) = −7.43, *p* = 3.18 × 10^−6^, unpaired *t* test).

**Fig. 6:**
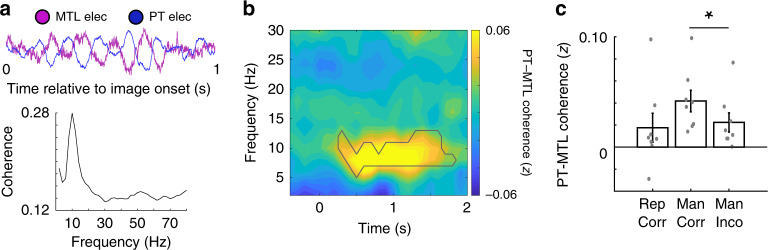
Alpha coherence increases between MTL and PT when recognizing manipulated images. **a** Representative iEEG traces from a PT and MTL electrode in an example participant indicating low frequency coherence at the single trial level. Across all trials in this electrode pair, coherence spectrum showed a peak in the low frequency band. **b** Average coherence spectrum for all PT-MTL electrode pairs across participants (black outline, *p* < 0.05, permutation procedure). **c** Average low frequency coherence between PT and MTL electrodes across eight participants for manipulated correct, repeated correct, and manipulated incorrect conditions. Greater alpha coherence for the manipulated correct condition compared to the manipulated incorrect condition was observed (*t*(7) = 2.54, *p* = 0.038, unpaired, two-tailed *t* test). All error bars indicate standard error of mean.

Successfully identifying whether an image has been manipulated during recognition testing requires comparing that image to a retrieved memory. Because our data suggest that retrieval may involve activation of the MTL, we were interested in whether neural communication between the MTL and the visual association cortex where differences were first detected might underlie this process. Low frequency oscillatory coherence has previously been linked with neural communication between brain regions^18–20^, and so we examined oscillatory coherence between electrode pairs in our data in participants with electrodes in multiple brain regions (see “Methods”). In a representative single trial, we observed clear evidence of phase-lagged alpha (8–12 Hz) oscillations, and consequently alpha coherence, between a pair of electrodes in the MTL and PT, suggesting that these two regions may become synchronized during the recognition period (Fig. 6a). We computed the coherence at all frequencies between all visually responsive PT and MTL electrodes during the recognition period across participants and found alpha coherence between the two regions significantly increased 300–1800 ms after the presentation of the image when examining all trials (*p* < 0.05, permutation procedure; Fig. 6b). We confirmed this by examining whether alpha synchrony alone, as opposed to coherence, increased after the presentation of the image (see “Methods”). We found significant increases in alpha synchrony during the same 300–1800 ms period after the presentation of the image compared to the 1 s period before image presentation (*t*(16) = 2.29, *p* = 0.028, one-tailed paired *t* test; Supplementary Fig. 12). Together, these data suggest that comparing past to present visual experience involves communication between the MTL and visual association cortex. We did not find evidence of significant coherence between the MTL and PAR (*p* > 0.05, permutation procedure).

We then examined coherence between PT and MTL electrodes separately for the manipulated correct, repeated, and manipulated incorrect conditions (Supplementary Fig. 13). Alpha coherence between these brain regions appeared more robust and more prolonged during the manipulated correct trials compared to the other conditions. We computed the mean level of alpha coherence over the recognition period and found significantly greater alpha coherence for the manipulated correct condition compared to the manipulated incorrect condition (*t*(7) = 2.54, *p* = 0.038, unpaired *t* test; Fig. 6c) and greater coherence when compared to the repeated condition (*t*(7) = 2.33, *p* = 0.05). The involvement of low frequency coherence raises the possibility that low and high frequency activity may be coupled, as suggested by recent evidence demonstrating such interactions in the MTL during successful memory updating^21^. We did not observe significant changes in theta- or alpha-high frequency coupling in the MTL across conditions (Supplementary Fig. 14).

## Discussion

**Fig. 7:**
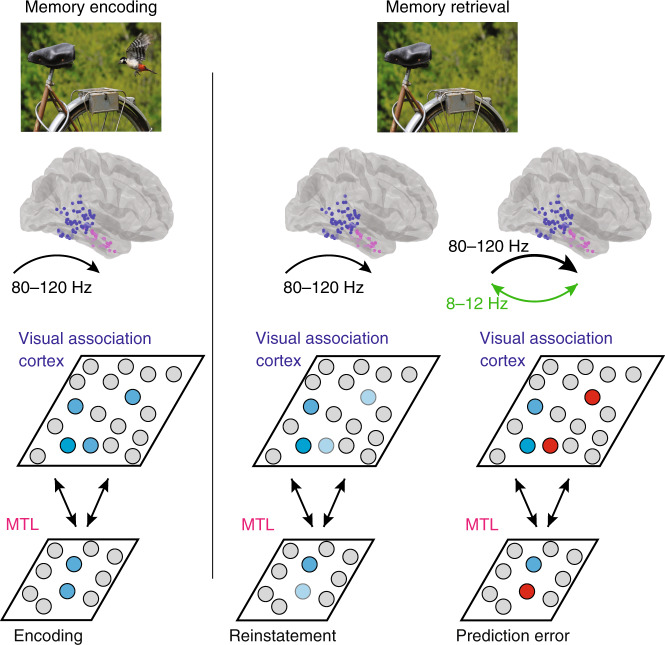
Model of cortical–medial temporal lobe interactions during visuospatial recognition. Initially viewing an image evokes image-specific 80–120 Hz activity within the visual association cortex and medial temporal lobe (MTL; blue units). During successful memory retrieval, the neural representation of a previously encoded image is reinstated within the visual association cortex and MTL. Successful reinstatement of this past experience is compared to current visual experience (dark and light blue units). In this case, the present visual experience no longer contains the bird that was in the remembered past visual experience. Any differences between present and past visual experience evoke an increase in 80–120 Hz power (bold arrow), which reflects an error signal, that arises in visual association cortex and propagates to the MTL (red units). This error signal is accompanied by low frequency coherence between these brain regions (green).

Our data demonstrate the neural dynamics that underlie how episodic memories may be used to compare present to past experience. Visually processing an image generates an 80–120 Hz signal that progresses from visual association cortex to the MTL and contains information regarding the specific image being viewed. Subsequently viewing the same image generates a similar rise in 80–120 Hz power, but this increase in 80–120 Hz power is larger and more prolonged when participants successfully recognize that the image has been manipulated. This excess signal, representing the difference between the present visual experience and the memory of the past experience, arises first in the visual association cortex before propagating to the MTL and is accompanied by low frequency oscillatory coherence between the two brain regions (Fig. 7).

Our findings therefore build upon previous evidence that neural activity is optimized to maximize information^1–3^ and extend this framework to episodic memories. Episodic memory formation relies upon feedforward and feedback interactions between the neocortex and the MTL^12–14,22^. Retrieving an experience stored in memory involves reactivating neural patterns of activity that were present during the original experience through similar interactions^15,16,23^. Memory retrieval therefore may enable the brain to generate an internal comparative model for each experience that can be used in order to optimize the information captured from each new experience.

We interpret our results to suggest that the ability to detect whether a new visual experience differs from these internal models relies upon episodic memory. Whether episodic memory is truly engaged in this process, however, requires careful consideration. We base this interpretation on several aspects of our task. Participants are not immediately tested on each individual image but must retrieve its memory following the presentation of several other intervening images and attention diverting tasks, such as locating changes in other images. In addition, each image to be remembered is a natural scene containing several items and complex associations. The number of total items to be remembered within each list therefore likely exceeds the capacity of working memory and is not amenable to simple rehearsal. These factors together suggest that performance on this task likely draws upon episodic memory. In our task, however, participants are tested on the memory of the images immediately after the study period. Hence, whether these results would generalize to longer-term memory retrieval over the span of several days or weeks is unclear, and it is possible that, after a prolonged period of memory consolidation, prediction error signals related to memory may arise first in a different brain region.

Regardless of the specific type of memory deployed here, our results demonstrate that, after encoding a series of visual images in memory, participants use the memory of these images to identify whether subsequent images have been manipulated. We find that 80–120 Hz power increases when participants are able to successfully identify these manipulations, suggesting that this activity reflects the difference between the present visual experience and a past experience stored in memory. Consistent with this interpretation, we find that this increase in activity is temporally locked to when the participants gaze at the region of the image where the manipulation occurs. Importantly, we find that this signal emerges when a manipulation involves both adding and removing an item from the image. This activity therefore reflects the detection of a change in the image as compared to memory rather than additional visual inputs from the new image.

An alternative explanation for our data is that the observed increases in 80–120 Hz power may simply reflect greater neural activity associated with the non-overlapping representations of the past and present experience rather than an explicit comparison between them. In this scenario, both the past and present visual experiences could activate different neural representations that collectively would appear as an overall increase in activity. Conversely, if the present experience were identical to the past experience, only one neural representation would be active, resulting in overall lower activity than the manipulated condition. While we cannot rule out this possibility, devoting entirely different neural representations to past and present visual experience would appear to be computationally inefficient and would violate the hypothesis that neural activity is optimized to maximize information. In addition, in our task, successfully identifying that a manipulation has occurred requires identifying whether items have been added or removed from the previous image stored in memory. Without an explicit comparison between the present and past experience, it is unclear how non-overlapping representations of past and present experience may lead to the same profile of activation during these two different conditions.

If episodic memories are used to establish predictions for future experiences, then one possibility could be that such comparisons are first generated in the MTL. Activity within the MTL has been intimately linked with both episodic memory formation and retrieval and implicated in rapid one shot learning^12–14^. Indeed, during memory retrieval, we find a rapid feedforward propagation of 80–120 Hz power from visual association areas to the MTL. However, while overall MTL activation occurs early during recognition, we find that the specific differences in 80–120 Hz power that emerge when participants successfully identify a manipulation in the visual images as compared to incorrect or repeated trials arise first in the visual association cortex before subsequently arising in the MTL. Our data therefore suggest that the internal models established by episodic memory are represented as early as visual association cortex, typically associated with higher-order processing of complex visual scenes. We find that the visual association cortex is capable of making such comparisons between the current visual experience and previous visual memories, consistent with recent proposals suggesting that each brain region is capable of performing computations on the representations contained therein^24^.

Our data are therefore consistent with and extend the principles of predictive coding to episodic memory. This hypothesis posits that one of the main functions of the brain is to generate an internal model of the world in order to predict future external input^4,5,7,25^. Under this framework, higher-order neocortical circuits send feedback to lower-order neocortical circuits in order to predict external sensory input and to reduce any redundancies of information when processing new stimuli. Differences between the predicted and actual inputs are transmitted from lower-order areas to higher-order areas through feedforward interactions in order to improve this internal model^5,6^. Empiric support for the principles of predictive coding derives from observations that have been made within lower-level sensory circuits. For example, surround suppression within the retina and the receptive field properties of end-stopping cells in primary visual cortex are canonical examples of how neural activity reflects a comparison between an external input and expectations of that input based on an internal model^2,5,26^.

One key distinction, however, between any internal model that is established for lower-level sensory inputs and those that may be used for episodic memory lies in how such models are generated. The internal models used to set our expectations for sensory stimuli are largely established based on a lifetime of observing and learning the statistics of the natural world^5,8,25,27^. If generating internal models and comparing these models to new experiences is a computationally efficient and generalizable approach for cortical processing, such principles should then extend beyond simple sensory features to more complex stimuli such as events or episodes. In contrast to simple sensory stimuli, however, episodes are often only experienced just once. Our data demonstrate that even just a single exposure to a complex visual scene is sufficient to establish an internal model that can be used for comparisons with future experiences and that this comparison involves increases in 80–120 Hz activity within the visual association cortex that propagate to the MTL.

The neural activity that we observe that captures the difference between past and present visual experience is centered in the 80–120 Hz band. This proposed role for 80–120 Hz activity is consistent with previous proposals for feedforward and feedback interactions in the primate visual system. The primate visual hierarchy consists of a set of cortical areas that exhibit neurophysiological asymmetries^17,18,28^. Feedforward interactions are characterized by higher frequency activity, while feedback interactions between these brain regions are mediated by lower frequency synchronization. Based on these physiological asymmetries, prediction errors are hypothesized to be transmitted in a feedforward direction by high frequencies while predictions are thought to be conveyed in a feedback direction through lower frequency activity^6,18^. The observed differences in 80–120 Hz power between past and present visual experience that propagate from the visual association cortex to the MTL are consistent with this hypothesis, as is the observed low frequency coherence between these brain regions that accompanies these difference signals and that may be involved in conveying predictive feedback. The increase in 80–120 Hz power observed when individuals are seeing an image for the first time during the encoding phase of the task is also consistent with this interpretation, as in that case the novel image is different from any previous experience the participant has had. However, although we find that both low and high frequency activity are involved in this process, we did not observe significant coupling between them that varied as participants successfully recognize a manipulated image. Further examining these interactions as prediction errors are generated based on memory would therefore remain an important topic of future investigation.

Together, our data therefore provide a direct account for how episodic memories can establish expectations that are used for comparisons in future experiences. Our results extend and support the hypothesis that one of the primary functions of the brain is to establish predictions based on its previous experiences. In this case here, our work shows that the internal models and predictions used to evaluate future experiences can be generated using episodic memories formed after just a single exposure. These single experiences are sufficient to establish a representation of a visual memory in the visual association cortex that is used for comparison with future visual experiences, thus suggesting that every experience that we encode into memory can be used to set our expectations and predictions for our future actions.

## Methods

### Participants

Fourteen participants (7 males; 40.9 ± 12 years) participants with drug-resistant epilepsy underwent a surgical procedure in which platinum recording contacts were implanted subdurally on the cortical surface as well as within the brain parenchyma. In each case, the clinical team determined the placement of the contacts to localize epileptogenic regions. The Institutional Review Board of the National Institutes of Health and the National Institute of Neurological Disorders and Stroke approved the research protocol used to acquire these data (protocol 11-N-0051), and informed consent was obtained from the participants and their guardians. All analyses were performed using custom built Matlab code (Natick, MA).

### Visuospatial recognition memory task

Participants performed a version of a visuospatial recognition task that has previously been used to identify individuals with early signs of memory impairment (Fig. 1a)^29,30^. During the encoding portion of the task, we sequentially presented participants a set of four color images. Each image was a natural scene containing between one and five items, such as an animal, person, or object, and we instructed the participants to remember each scene. We presented each image for 5 s, and a white fixation cross appeared for 1 s before each image. Immediately following the list of four images, we then presented the same images during the recognition testing portion of the task and tested the participants on their memory for the images. The images during recognition testing were presented in the same order as they were presented during encoding, thus ensuring that a lag of four images separated study and test of each image. The average time delay between when an image was presented during the encoding portion of the task and when that image was tested during the recognition portion of the task was 28 ± 6 s (mean ± SD) across participants.

Critically, we manipulated some of the images presented during recognition testing by either adding or removing an item from the scene. We therefore presented three different types of images during recognition testing—added, removed, or repeated—depending on whether a manipulation had been performed and the type of manipulation. Images that were repeated, that had items added, and that had items removed comprised 28, 36, and 36% of the testing trials, respectively. For most analyses, we considered added and removed trials together as the manipulated condition. We selected images from an open access database of images from Pixabay (Munchen, Germany) and Pexel (Fuldabruck, Germany). We used Adobe Photoshop (San Jose, CA) to remove an item from each of the original images. We used the image with the removed item as the image to be tested during recognition testing for the removed condition and as the image to be remembered during the encoding period for the added condition.

During recognition testing, participants viewed the images until they made their response. They indicated whether the image was the same or changed using left and right arrow keys. We divided the recognition trials into manipulated correct, manipulated incorrect, and repeated correct trials depending on whether there was a manipulation of the image and on the participant’s response. If the participant indicated the image was manipulated, we then presented a mouse cursor at the center of the screen and instructed the participant to identify the location of the manipulated item using a mouse click. We removed all trials in which the response time for identifying whether an image was manipulated >10 s. For each image, we defined a critical region as a rectangular region around each item. Based on where the mouse click fell within this rectangular region, we determined whether the participant was able to correctly identify the location of the manipulated item (Fig. 1d).

In this task design, each image studied by the participants contains 2–5 items, and therefore a list of 4 images contains approximately 8–20 items. We computed the number of items held in memory during each list using Cowan’s *K* formula, (*HitRate* + *CorrectRejectionRate* − 1) × *N*, where *N* is the memory set size^31^. Based on the observed hit rate and correct rejection rate, we approximated the number of items held in memory by the participants during each list (Cowan’s *K*) to range between 4.4 and 10.9. This range likely exceeds the capacity of working memory^31^, particularly given the complex images that are remembered in this task^32^.

Participants completed one to two sessions during the monitoring period. Each session was approximately an hour of testing and contained 60 lists of images, where each list contained the sequential presentation of four images during encoding and the same (or manipulated) four images during recognition testing. Participants completed a total of 784 ± 195 trials during the monitoring period.

### iEEG recordings

Depending on the amplifier and the discretion of the clinical team, iEEG signals were sampled at 1000 or 2000 Hz. For clinical visual inspection of the recording, signals were referenced to a common contact placed subcutaneously, on the scalp, or on the mastoid process. The recorded raw iEEG signals used for analyses were referenced to the system hardware reference, which was set by the recording amplifier (Nihon Kohden, Irvine, CA) as the average of two intracranial electrode channels. We re-referenced these raw signals using bipolar referencing (see below) in order to mitigate any effects of volume conduction or any biases introduced by the system hardware reference. All recorded traces were resampled at 1000 Hz, and a fourth-order 2 Hz stopband butterworth notch filter was applied at 60 Hz to eliminate electrical line noise.

We collected electrophysiological data from a total of 1716 subdural and depth recording contacts (122 ± 5.9 per participant; PMT Corporation, Chanhassen, MN). Subdural contacts were arranged in both grid and strip configurations with an inter-contact spacing of 10 mm. Contact localization was accomplished by co-registering the post-op CTs with the postoperative magnetic resonance imagings (MRIs) using both the FSL Brain Extraction Tool (BET) and FLIRT software packages and mapped to both Montreal Neurological Institute (MNI) and Talairach space using an indirect stereotactic technique and OsiriX 11.0 Imaging Software DICOM viewer package. The resulting contact locations were subsequently projected to the cortical surface of a MNI N27 standard brain^33^. Preoperative MRIs were used when postoperative MRIs were not available.

We divided projected electrode contacts into four regions of interests based on their location relative to the Desikan–Killiany atlas^34^: LOC, PAR, PT, and MTL. We assigned all electrodes with locations in the lateral occipital lobe as LOC electrodes, and all electrode contacts in the superior and inferior PAR lobe as PAR electrodes. We identified all electrodes with locations in posterior part of the superior, middle, or inferior lateral temporal cortex or that lay over the posterior aspect of the fusiform gyrus as PT electrodes^35^. We designated all depth electrode contacts within the hippocampus and all subdural contacts that lay along the MTL structures, including the parahippocampal gyrus and the entorhinal cortex, and that were medial to the collateral sulcus as MTL electrodes. Across participants, the average number of electrodes assigned to the LOC, PIT, PAR, and MTL were 3 ± 2, 21 ± 35, 8 ± 5, and 13 ± 9 electrodes, respectively.

To identify changes within different substructures of the MTL, we further divided MTL electrodes into parahippocampal gyrus, entorhinal cortex, and hippocampus. We used the Desikan–Killany atlas to identify electrodes that overlay the parahippocampal gyrus and entorhinal cortex, and we identified hippocampal depth electrodes using FSL’s automated subcortical segmentation package. We excluded hippocampal depth electrodes that were also localized to the parahippocampal gyrus according to the Desikan–Killiany atlas. In our data, the parahippocampal gyrus, entorhinal cortex, and hippocampus contain electrodes from the 9, 8, and 5 participants, respectively. For these participants, the number of electrodes within each of these regions is 7.4 ± 7.5, 5.9 ± 3.6, and 5.4 ± 2.1, respectively (mean ± SD).

We analyzed iEEG data using bipolar referencing to reduce volume conduction and spurious signals introduced by the system reference. The choice of referencing largely depends on the assumptions regarding the spatial distribution of the signal of interest^36^. Bipolar referencing offers a practical approach for examining local events with spatial distributions that are smaller than the inter-electrode distance of our recordings since it will filter out activity at the larger spatial scale that is common to both electrodes that may be introduced by the system reference or that may result from volume conduction. In addition, because each neighboring bipolar channel records activity from similar brain regions through similar electrode contacts, bipolar referencing ensures that any referencing that is applied to each recorded iEEG trace is performed using a reference electrode that shares similar impedance and noise profiles. Finally, bipolar referencing has also been noted to be superior to the average reference montage in reducing muscular artifacts in iEEG^37^. We defined the bipolar montage in our data set based on the geometry of iEEG electrode arrangements. For every grid and strip, we isolated all pairs of contacts that were positioned immediately adjacent to one another. Bipolar signals were then calculated by finding the difference in the signal between each pair of immediately adjacent contacts. The resulting bipolar signals were treated as new virtual electrodes (henceforth referred to as electrodes throughout the text), originating from the midpoint between each contact pair. All subsequent analyses were performed using these derived bipolar signals.

High frequency activity can be associated with epileptiform activity in addition to cognitive processes. Therefore we implemented several measures to provide the most conservative sampling of non-pathological signals possible. We implemented an automated trial and electrode rejection procedure based on excessive kurtosis or variance of iEEG signals^35^. We calculated and sorted the mean iEEG voltage across all trials, and divided the distribution into quartiles. We identified trial outliers by setting a threshold, Q3 + w*(Q3 − Q1), where Q1 and Q3 are the mean voltage boundaries of the first and third quartiles, respectively. We empirically determined the weight *w* to be 2.3. We excluded all trials with mean voltage that exceeded this threshold. The average percent removed across all sessions in each participant due to either system-level noise or transient epileptiform activity was 1.7 ± 0.2% of all electrodes and 2.8 ± 0.1% of all trials.

In addition to system level line noise, eye-blink artifacts, sharp transients, and inter-ictal epileptiform discharges (IEDs) can confound the interpretation of our results. We therefore implemented an automated event-level artifact rejection^38^. We calculated a *z*-score for every iEEG time point based on the gradient (first derivative) and amplitude after applying a 250 Hz high pass filter (for identification of epileptogenic spikes). Any time point that exceeded a *z*-score of 5 with either gradient or high frequency amplitude was marked as artifactual, and 100 ms before and after each identified time point was also classified as an artifact. We visually inspected the resulting iEEG traces and found that the automated procedure reliably removed IEDs and other artifacts. In total, following bipolar referencing and exclusion of electrodes because of artifact, our pre-processed data set consisted of 1716 bipolar electrodes (123 ± 5.9 per participant).

### Eye movement and fixation detection

In a subset of participants (*n* = 8), we tracked the locations of their gaze on the screen using a Tobii X3-120 EyeTracker (Stockholm, Sweden) that sampled eye movements at 120 Hz. At the start of each session, participants performed a calibration procedure in order to convert eye rotations into a set of gaze positions relative to the screen. For each participant, we extracted raw eye movement data during the experimental session and converted the movements into a set of fixations using a dispersion-based algorithm^39^. We defined each fixation point as a point on the screen upon which gaze continually remained within 2 degrees of visual angle for a period of ≥100 ms. We excluded the remaining six participants from the analysis due to the inability to calibrate the participants or collect eye movement data due to clinical constraints.

### Spectral power

We quantified spectral power and phase by convolving iEEG signals with complex valued Morlet wavelets (wavelet number 6). We extracted data from all encoding and recognition trials, beginning with the presentation of the image on the screen until the image was removed during encoding or until the response during recognition testing and localization, for our analyses. In all trials, we included a 1000 ms buffer on both sides of the clipped data. To generate corresponding power spectrograms, we calculated spectral power using 32 logarithmically spaced wavelets between 2 and 431 Hz. We then squared and log-transformed the continuous time wavelet transform to generate a continuous measure of instantaneous power. To account for changes in power across experimental sessions, we *z*-scored power values separately for each frequency and for each session using the mean and SD of all respective values for that session. We binned the continuous time *z*-scored power for each frequency into 200 ms epochs spaced every 100 ms (50% overlap) and averaged the instantaneous power over each epoch and performed subsequent analyses on these binned values.

To control for the number of trials across conditions for spectral power analysis, we used a bootstrap procedure. We randomly subsampled trials from the condition with the larger number of trials to match the number of trials in the smaller condition. We repeated this procedure 100 times and calculated the average spectral power during each iteration. We assigned the average value of these iterations as the final value for the higher-trial-count condition and used this average bootstrapped value for comparison with the lower-trial-count condition.

### Metrics of reinstatement

To quantify reinstatement of representations during the recognition period, we conducted a representational similarity analysis^15,40^. Briefly, we binned the continuous time *z*-scored power for each frequency into 200 ms epochs spaced every 100 ms (50% overlap) and averaged the instantaneous power over each epoch. For each temporal epoch, we subsequently averaged the *z*-scored power within each frequency band. For every temporal epoch in each trial, we constructed a feature vector composed of the average *z*-scored power for every electrode within a given region of interest and for each frequency band. For each encoding temporal epoch, *i*, and for each retrieval temporal epoch, *j*, we define feature vectors as follows:

$$\begin{array}{l}{\overrightarrow{E}}_{i}=[{z}_{1,1}(i)\,\ldots \ {z}_{1,F}(i)\,\ldots \ {z}_{L,F}(i)],\\ {\overrightarrow{R}}_{j}=[{z}_{1,1}(j)\,\ldots \ {z}_{1,F}(j)\,\ldots \ {z}_{L,F}(j)],\end{array}$$

where *z*_*l*,*f*_(*i*) is the *z*-transformed power of electrode *l* = 1…*L* at frequency band *f* = 1…*F* in temporal epoch *i*. For *L* electrodes and *F* frequency bands, we thus create a feature vector at each temporal epoch that contains *K* = L*F features, which represents the distributed spectral power across all electrodes and across all frequency bands. In our main analysis, we focus on the patterns of neural reinstatement within a single frequency band centered at 80–120 Hz. This frequency band corresponds to the main changes in spectral power observed in response to the presentation of an image (Fig. 2c). In subsequent analyses, we also found that the greatest changes between conditions were centered in this 80–120 Hz frequency band (Supplementary Fig. 5). This frequency band is also consistent with previous work suggesting the presence of high frequency 80–120 Hz ripples in the human cortex that are relevant for memory retrieval^16^. Thus, in our main analysis, we construct a feature vector containing just this single frequency band and therefore containing only *K* = *L* features.

To quantify reinstatement during trial *n*, we calculated the cosine similarity between encoding and recognition feature vectors ${\overrightarrow{E}}_{i}$ and ${\overrightarrow{R}}_{j}$ for all pairs of encoding and recognition temporal epochs during that trial. Cosine similarity gives a measure of how close the angles of two vectors are in a multidimensional space. We chose cosine similarity over Pearson’s correlation to measure reinstatement because, if all of the elements of two feature vectors show increases in power from baseline, with small additional random noise, then these two vectors should have high measured reinstatement. Pearson’s correlation, a centered version of cosine similarity, would give a low correlation in this case because of the noise fluctuations, whereas the cosine similarity would be high, consistent with our interpretation of reinstatement. Thus, for each trial, *n*, we generate a temporal map of reinstatement values:

$${C}_{n}(i,j)=\frac{{\overrightarrow{E}}_{i}\cdot {\overrightarrow{R}}_{j}}{\parallel {\overrightarrow{E}}_{i}\parallel \parallel {\overrightarrow{R}}_{j}\parallel },$$

where *C*_*n*_(*i*, *j*) corresponds to the reinstatement of neural activity across all electrodes and all frequencies between encoding epoch *i* and retrieval epoch *j* during trial *n*. We *z*-scored the reinstatement maps by the average cosine similarity across all trials to produce a normalized reinstatement map. We averaged the normalized reinstatement maps separately across trials for each participant and averaged the reinstatement maps across participants.

To confirm that the reinstatement of neural activity was specific to each image, we compared the true reinstatement to reinstatement computed after shuffling the trial labels. We shuffled the encoding–retrieval trial pairs for all trials within each list of images. In this manner, we compared the pattern of neural activity present in an individual retrieval trial to the neural activity present in a non-matching encoding trial. We computed the average shuffled reinstatement across all trials and then compared this average to the average of the true encoding–retrieval reinstatement in each participant (Fig. 2e). If neural activity is specific to an image, the difference between the true reinstatement and reinstatement using the shuffled encoding–retrieval trial pairs should be greater than zero (see “Statistical analysis”).

To examine reinstatement within individual brain regions, we followed the same procedure above, but in this case constructed feature vectors using only the subset of electrodes within each of the regions of interest. For each participant, we calculated the normalized image-specific reinstatement strictly using electrodes within a particular region and then assessed for significance across participants using the same shuffling procedure (Supplementary Fig. 2a). We also computed a mean time series of reinstatement during retrieval for each region of interest. To do so, we identified all the encoding time epochs in the reinstatement map that exhibited significant image-specific reinstatement across participants. For each participant, we generate a time series of reinstatement during retrieval by computing the mean reinstatement across these significant encoding epochs for every time point during retrieval. We then calculated the average time series of reinstatement for each region across participants (Fig. 2f)

### Generation and characterization of cross-correlograms

We computed a cross-correlation of the spectral power time series between pairs of electrodes spanning PT and MTL and spanning PAR and MTL in order to examine the temporal relation of high frequency activity between visual association cortex and MTL. We used the first second of data following image presentation during recognition testing for this analysis. In each electrode, we extracted the continuous time series of spectral power and divided this trace into non-overlapping 10 ms bins by averaging the time series over each bin. We used these 10 ms bins to compute the cross-correlations for computational efficiency and to generate more temporally smoothed representations of the cross-correlations between electrode pairs. For every electrode pair, in each trial, we computed the time-lagged cross-correlation between the time series of binned values. We then averaged these cross-correlations across trials, thus generating a true cross-correlogram for each pair of electrodes in each participant that we can compare to a chance distribution and that we can use to identify the time lag of high frequency activity between the electrode pair.

We generated a chance cross-correlogram for each electrode pair characterizing the baseline cross-correlation that would be expected by chance given the presentation of a stimulus^16^ and to which the true correlogram could be compared. For every pair of electrodes, we generated this chance distribution by computing the cross-correlation of the power time series of one electrode during a randomly chosen individual trial with the time series of the other electrode from another randomly chosen trial. We repeated this procedure 100 times and averaged across all permutations to generate an average chance cross-correlogram for that electrode pair. The difference between the true cross-correlogram and the chance cross-correlogram reflects the extent to which two signals are cross-correlated greater than chance given the presentation of a stimulus.

To assess significant coupling for a single electrode pair, we compared the true distribution of cross-correlation values between −50 and 50 ms to the chance distribution in this same window using a paired *t* test. To assess significant coupling between two regions across participants, we first averaged the true and chance cross-correlation values over this window for each electrode pair and then computed the average difference between the true and chance cross-correlograms across all electrode pairs between two regions for a single participant. We then compared the distribution of these average differences across participants to 0 to assess significance (*p* < 0.05, paired *t* test). To determine the relative timing of high frequency power between two regions, we identified the peak time of each correlogram for every pair of electrodes between two regions. We computed the average peak time across electrode pairs between the two regions for each participant and assessed whether the distribution of average peak times across participants was significantly different than zero (*p* < 0.05, paired *t* test).

### Temporal dynamics of spectral power

We compared the peak times of the spectral power time series for LOC, PT, PAR, and MTL in order to examine the temporal relation of high frequency activity through the visual hierarchy. We identified the peak time for a particular region by calculating the time during which the average spectral power time series across participants reached its maximum value. We then computed the difference in the peak times between two regions to identify the temporal relation of spectral power between them. To assess whether this difference was significant, we generated a chance distribution to which the true difference in peak times could be compared. We computed this chance distribution by randomly switching the spectral power time series for one region with the spectral power time series of the other region in each participant. Hence, in each permutation, some participants would retain their original power time series traces in their original regions, and some participants would have the labels for the regions randomly switched. We then averaged these shuffled spectral power time across participants for each region and then computed the difference in peak times between the two regions in each permutation. We repeated this procedure 1000 times to generate a shuffled distribution of differences in peak times. We assigned *p* values that characterize the difference in peak times between any two brain regions by comparing the true difference in peak times to the shuffled distribution of differences.

To estimate how quickly high frequency activity increased in each brain region and how this compared across LOC, PT, PAR, and MTL, we computed the instantaneous slope of the increases we observed in the time series of high frequency spectral power. We computed the difference in spectral power between adjacent time bins (200 ms overlapping bins incremented by 100 ms) and then averaged these estimates of instantaneous slopes across all time points within the first 500 ms after image presentation. Within each brain region in each participant, we computed the average instantaneous slope across all visually responsive electrodes. We compared the distribution of average values across participants between two brain regions using an unpaired *t* test (*p* < 0.05) in order to assess whether the rise in high frequency activity was different between the regions across participants.

To determine whether the differences in high frequency 80–120 Hz spectral power that we observed between conditions arose at different times in different brain regions, we performed two analyses. In both cases, we explicitly generated a time course of the average difference between conditions for each electrode that showed any significant difference between conditions in each brain region in each participant. In the first analysis, we used the rise in the average time series across all significant electrodes of the differences in spectral power to estimate the first time point when this difference deviates from zero and to estimate the time point when the increase in high frequency power reached 50% of its peak. We used this approach to generate a more temporally precise estimate of when this signal first increased above baseline since in our main analysis we generated the time series using overlapping 200 ms bins incremented every 100 ms. To estimate this initial time of deviation, we identified the time point of the peak difference between conditions and the time point of the local minimum that immediately preceded the peak difference. We then fit a line using all points in between these two time points and identified the time point when that line intersected with zero. We designated this as the time point at which the difference between conditions first deviates from baseline. We compared the distribution of these first time points across participants between brain regions using an unpaired *t* test. We similarly identified when the rise of spectral power reached 50% of the peak and compared the distribution of 50% time points across participants between each brain region. We also compared the estimated time points at which we first observed a rise in the difference in high frequency power between conditions to the time points at which we observed overall increases in high frequency power in the MTL across conditions. In a similar manner, we used the average time series of spectral power across significant MTL electrodes to estimate the first time point when overall 80–120 Hz power deviated from baseline in the MTL. In the second analysis, we identified the time points that exhibited the first significant difference in spectral power between conditions in each electrode within a region. We then averaged these first time points across all electrodes within each region in each participant. We compared the distribution of these time points of first differences across participants between brain regions (unpaired *t* test, *p* < 0.05).

### Spectral coherence

We computed the magnitude squared spectral coherence between every electrode pair using one second temporal epochs during the recognition period (MATLAB function “mscohere”)^41^. We computed the coherence between individual electrode contacts, rather than between bipolar virtual contacts, since bipolar referencing has been shown to remove low frequency coherence between iEEG electrodes^42^. In this case, before computing coherence between any electrode pair, we re-referenced the signal from each electrode to a global common average in order to eliminate common mode signals that would arise from the system-level reference or from artifacts. We calculated the coherence between two time series, *x*(*t*) and *y*(*t*), in two electrode contacts as a function of frequency:

$$C(f)=\frac{| {P}_{XY}(f){| }^{2}}{{P}_{XX}(f){P}_{YY}(f)},$$

where *P*_*XX*_ and *P*_*YY*_ are the power spectral densities and *P*_*XY*_ is the cross-spectral density. We generated a coherence spectrum for each temporal epoch, frequency, electrode pair, and trial. We then *z*-scored coherence values separately for each electrode pair using the mean and SD of all coherence values for the session. For any pair of brain regions, we restricted our analysis to only those participants with electrodes in both brain regions (*n* = 8 for PT–MTL coherence and *n* = 5 for PAR–MTL coherence).

### Phase synchrony

To confirm that any increases in coherence are not confounded by changes in oscillatory power, we obtained an estimate of inter-electrode phase synchrony. To do so, we first extracted the instantaneous phase of the complex valued Hilbert transform of the filtered signal in each frequency band. For every frequency band, *f*, and time point, *t*, we calculated a phase locking value (${\overline{R}}_{pq}$) between the continue phase series of two electrodes, *ϕ*_*p*_(*t*, *f*) and *ϕ*_*q*_(*t*, *f*)^43^:

1 $${\overline{R}}_{pq}(f)=\frac{1}{N}\left| \mathop{\sum }\limits_{t = 1}^{N}{e}^{i({\phi }_{p}(t,f)-{\phi }_{q}(t,f))}\right|,$$

where *N* is total number of samples collected from all trials at each time point. For each electrode, we obtained a normalized time series of phase locking values by *z*-scoring each value by the mean and standard deviation across all time points.

### Statistics and reproducibility

We employed a non-parametric clustering-based procedure to identify significant time, frequency, or time–frequency epochs for differences in power, coherence, and reinstatement between conditions^44^. The procedures for all analyses were identical with the exception that clusters identified for coherence and reinstatement analysis were generated across the two dimensions. The clustering procedure identifies contiguous temporal or time–frequency clusters exhibiting significant differences between two conditions (e.g., manipulated correct and repeated correct), with the null hypothesis that, across participants, each epoch showed no difference between the conditions. For each time or time–frequency window, we computed the true *t* statistic and *p* value across participants between the two conditions by comparing the distribution of average values across all visually responsive electrodes within each brain region across participants. The *p* value for each individual time point, time–frequency window, or time–time window in the true case, however, does not take into account the multiple comparisons that are made across time points.

To correct for multiple comparisons across time points, we randomly permuted the participant-specific averages between the two conditions. In practice, this translates to randomly reversing the sign of the difference within each participant and recomputing the mean difference across participants. For *n* participants, this results in an empiric distribution of 2^*n*^ possible mean differences that are all equally probable under the null hypothesis. We generated the empiric distribution from 1000 permutations for every time point and calculated *t* statistics for each time point in each permutation. We identified clusters containing time points or time–frequency windows that were adjacent in time (or in time–frequency space) that exhibited a significant difference between trial types (where in each time point, *p* < 0.05 unless specified otherwise) in both the true case and in each permutation. For each cluster of significant time points identified in the true and permuted cases, we defined a cluster statistic as the sum of the *t* statistics within that temporal cluster. We retained the maximum cluster statistic during each of the 1000 permutations to create a distribution of maximum cluster statistics. We assigned *p* values to each identified cluster of the true data by comparing its cluster statistic to the distribution of maximum cluster statistics from the permuted cases. Clusters were determined to be significant if their *p* value calculated in this manner was <0.05.

We used a similar procedure to identify electrodes showing a significant difference between conditions. The clustering procedure identifies contiguous temporal clusters exhibiting significant differences between two conditions with the null hypothesis that, across *trials*, each epoch showed no differences between the participants. In this case, we created a permuted distribution by randomly switching the condition of one trial with the condition of another and then computing the mean difference between the conditions. We repeated this procedure 1000 times to create an empiric distribution and compared the true difference to the empiric distribution of mean difference. We then identified the maximum cluster statistic of the true data and compared to the cluster statistic of the permuted distribution to correct for multiple comparisons. Clusters were determined to be significant if their *p* value calculated in this manner was <0.05.

Although we performed all analyses on discrete time windows, we used MATLAB’s *contourf* function to visualize isolines of the matrix of reinstatement or coherence values. This function fills the corresponding isolines based on a colormap. The time regions of significance, however, are based on the discrete time windows. We used the *contour* function to overlay the single isoline indicating the regions of significance.

All experiments and data analyses in this study were performed once and were not reproduced in a separate cohort of participants.

### Reporting summary

Further information on research design is available in the Nature Research Reporting Summary linked to this article.

## Supplementary information

Supplementary Information

Reporting Summary

## Data Availability

The source data sets generated during and/or analyzed during the current study can be found at https://neuroscience.nih.gov/ninds/zaghloul/downloads.html.

## Code availability

Custom MATLAB analysis code is available upon request.
