# Supplementary material for: Feedforward prediction error signals during episodic memory retrieval
Source: Nat Commun. 2020 Nov 27;11:6075. doi: 10.1038/s41467-020-19828-0 (PMC7699639; doi:10.1038/s41467-020-19828-0)
Supplement: Supplementary file 1 — Supplementary Information [file 41467_2020_19828_MOESM1_ESM.pdf]

Supplementary Information

Rafi U. Haque[1], Sara K. Inati[2], Allan I. Levey[3], and Kareem A. Zaghloul[1]

[1]*Surgical Neurology Branch, NINDS, National Institutes of Health, Bethesda, MD 20892, USA*
[2]*Office of the Clinical Director, NINDS, National Institutes of Health, Bethesda, MD 20892, USA*
[3]*Department of Neurology, Emory University, Atlanta, GA 30322, USA*

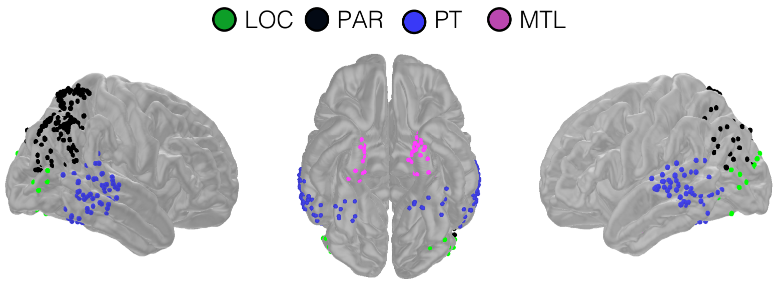

Figure S1 Electrode distribution in visual areas across subjects. All electrodes designated as within lateral occipital complex (LOC), posterior temporal(PT), parietal (PAR), and medial temporal lobe (MTL).

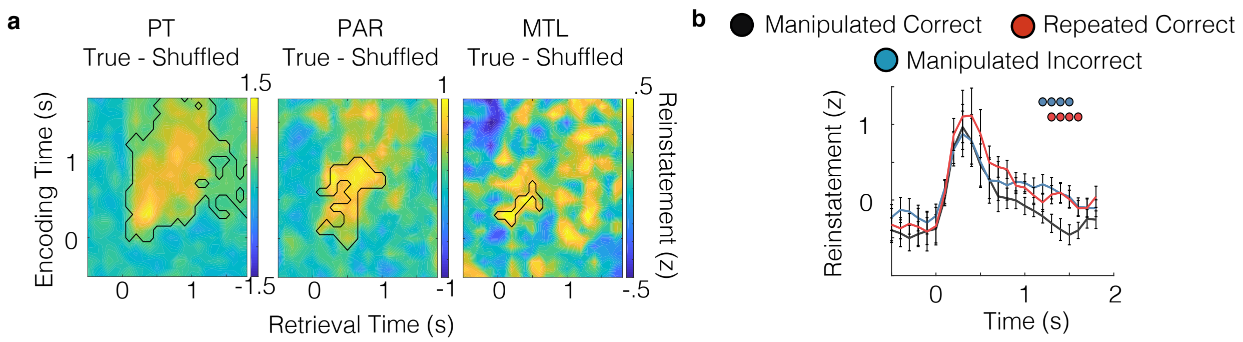

Figure S2 Differences in image-specific reinstatement by region and between conditions **a)** Image-specific reinstatement of 80-120 Hz power within PT, PAR, and MTL averaged across all participants. The difference in average reinstatement between the true and shuffled distributions reflects image-specific reinstatement across all participants (black outline, $p < .05$, permutation procedure). **b)** Time series of reinstatement across 14 participants for the manipulated correct, manipulated incorrect, and repeated correct conditions during the recognition period. Dots indicate significant decreases in reinstatement corrected for multiple comparisons ($p < .05$, permutation procedure; image appears at $t = 0$). All error bars indicate standard error of mean.

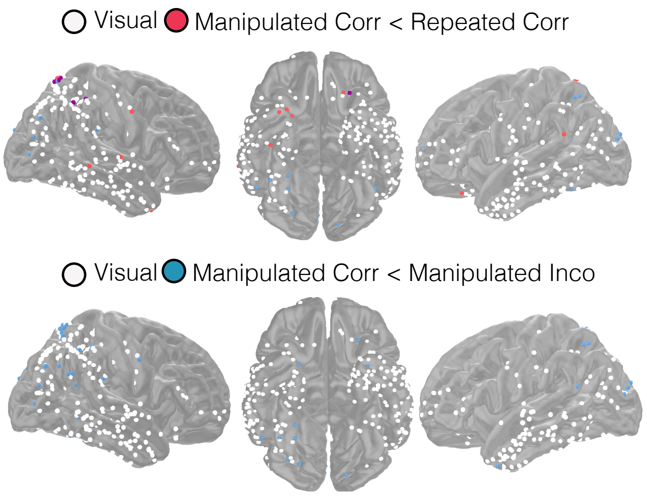

Figure S3 Decreases in 80-120 Hz power between conditions. Visually responsive electrodes (white) that showed significant decreases in 80-120 Hz power for the manipulated correct compared to the manipulated incorrect (blue), manipulated correct compared to the repeated correct (red), or both (purple).

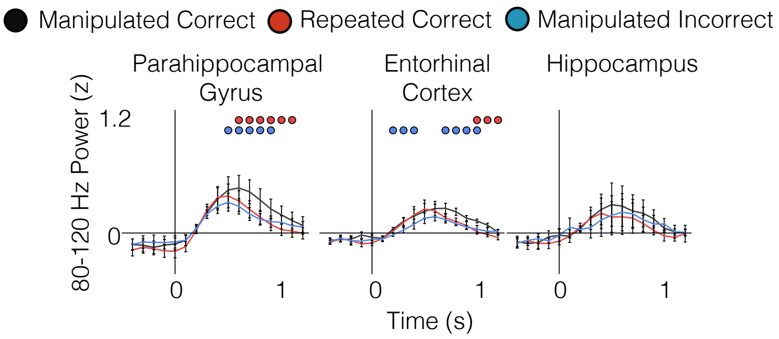

Figure S4 Differences in 80-120 Hz power within MTL regions between present and remembered visual experience. Time courses of 80-120 Hz power for the manipulated correct, manipulated incorrect, and repeated correct conditions locked to image presentation across 9 parahippocampal, 8 entorhinal, and 5 hippocampal participants during recognition (200 ms sliding windows, 50% overlap; image appears at $t = 0$). Blue and red dots indicate significant differences in 80-120 Hz power between manipulated correct and manipulated incorrect and repeated correct condition, respectively ($p < .05$, permutation procedure). All error bars indicate standard error of mean.

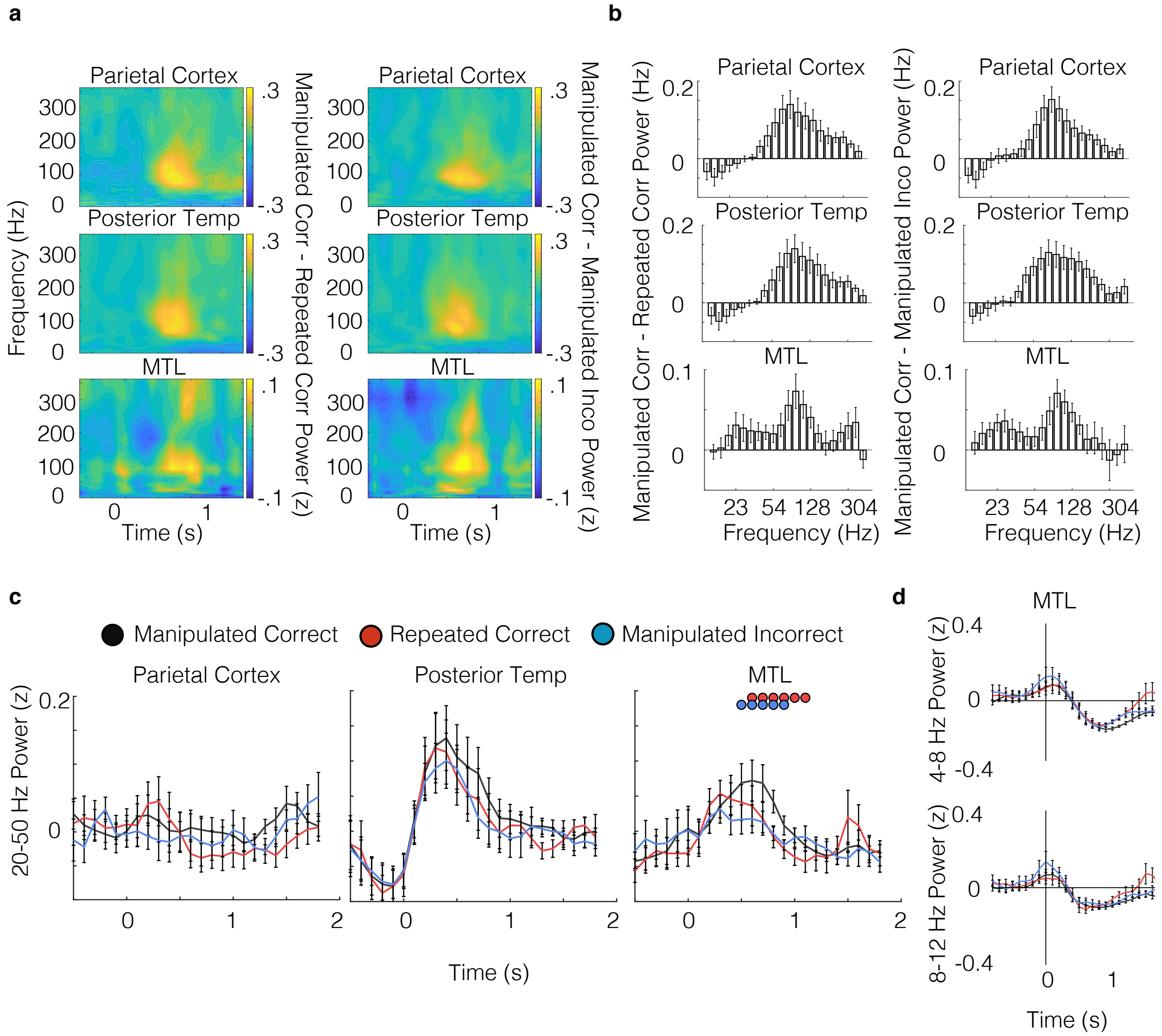

Figure S5 Differences in power between present and remembered visual experience across a range of frequencies. **a)** Spectrograms of power differences between the manipulated correct and manipulated incorrect and repeated correct conditions across participants in PAR, PT, and MTL.**b)** Average difference between the manipulated and repeated correct trials across participants for each high frequency **c)** Time courses of 20-50 Hz power across participants for the manipulated correct, manipulated incorrect, and repeated correct conditions in PAR, PT, and MTL during the recognition period (200 ms sliding windows, 50% overlap; image appears at $t = 0$). Blue and red dots indicate significant differences in 20-50 Hz power between manipulated correct and manipulated incorrect and repeated correct condition, respectively ($p < .05$, permutation procedure). All error bars indicate standard error of mean. In contrast to the PAR and PT, MTL demonstrated a peak in the 20-50 Hz band when comparing the manipulated correct to the manipulated incorrect and repeated correct conditions from 600 to 900 ms ($p < .01$, permutation test). **d)** Low frequency power within the MTL did not differ between conditions. Statistical analyses were conducted using 8 PAR, 10 PT, and 10 MTL participants.

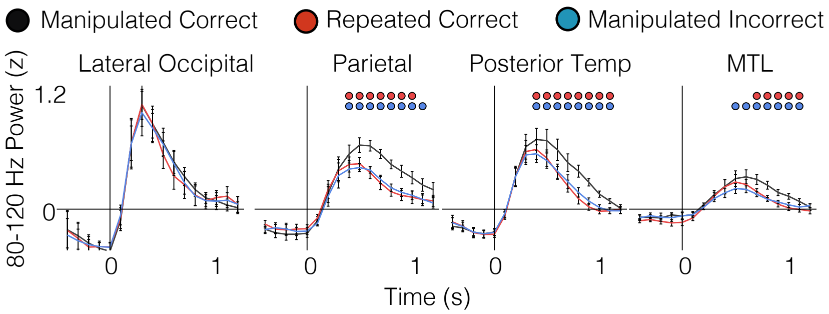

Figure S6 Increases in 80-120 Hz power in visual association cortex and MTL reflect differences between present and remembered visual experience after correction for trial counts. Time courses of 80-120 Hz power for the manipulated correct, manipulated incorrect, and repeated correct conditions locked to image presentation across 6 LOC, 8 PAR, 10 PT, and 10 MTL participants during recognition (200 ms sliding windows, 50% overlap; image appears at $t = 0$). Trial counts were equated across conditions using a bootstrap procedure.Blue and red dots indicate significant differences in 80-120 Hz power between manipulated correct and manipulated incorrect and repeated correct condition, respectively ($p < .05$, permutation procedure). All error bars indicate standard error of mean.

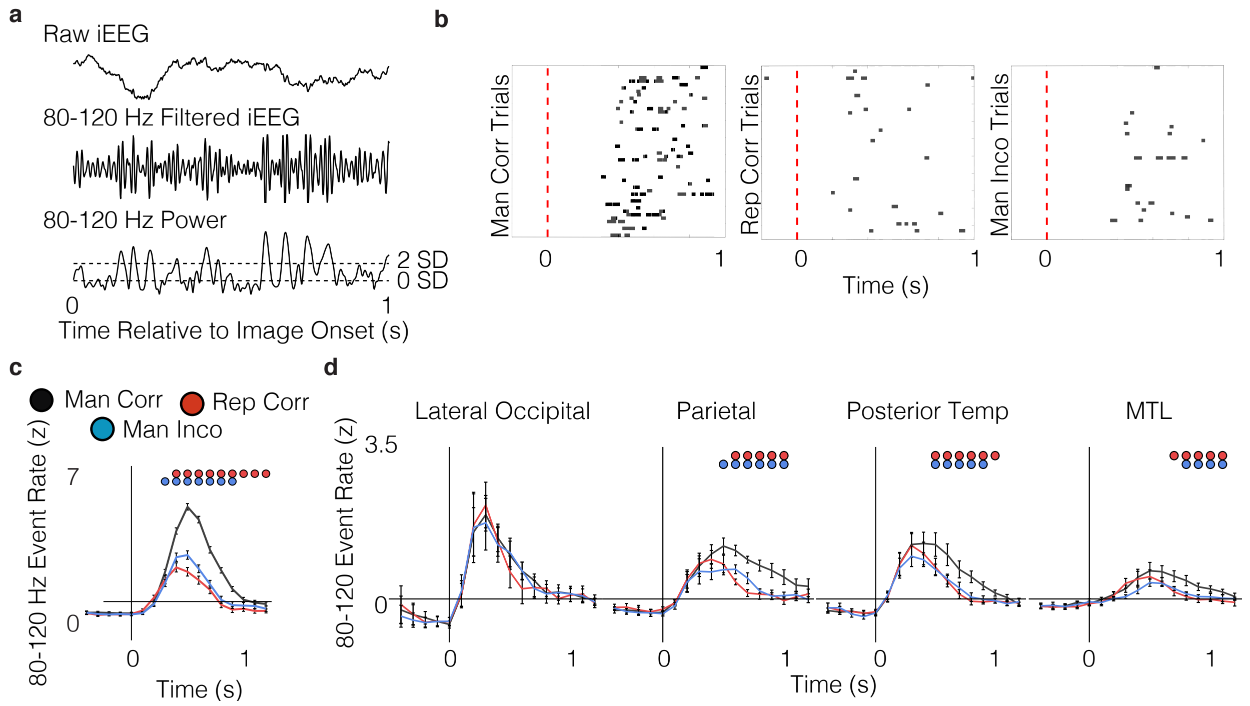

Figure S7 Discrete 80-120 Hz events increase when recognizing differences between present and remembered visual experience. **a)** Raw and 80-120 Hz filtered iEEG signal filtered. We identified discrete 80-120 Hz events as time points where the Hilbert amplitude exceeded two standard deviations above the mean amplitude of the filtered traces for 20 ms. Previous evidence has demonstrated that discrete 80-120 Hz ripples are associated with successful memory retrieval[1]. **b)** Rasters plots of 80-120 Hz events in a representative electrode in PT across manipulated correct, manipulated incorrect, and repeated correct condition. **c)** Average event rate for a PT electrode across 207 manipulated correct (black), 95 manipulated incorrect (blue), and 94 repeated correct (red) recognition trials (200 ms sliding window, 50% overlap; image appears at $t = 0$). Blue and red dots indicate significant differences in 80-120 Hz power between manipulated correct and manipulated incorrect and repeated correct condition, respectively ($p < .05$, permutation procedure). **d)** Time courses of 80-120 Hz event rate across participants for the manipulated correct, manipulated incorrect, and repeated correct conditions across 6 LOC, 8 PAR, 10 PT, and 10 MTL participants during the recognition period (200 ms sliding windows, 50% overlap; image appears at $t = 0$). Blue and red dots indicate significant differences in 80-120 Hz power between manipulated correct and manipulated incorrect and repeated correct condition, respectively ($p < .05$, permutation procedure). All error bars indicate standard error of mean.

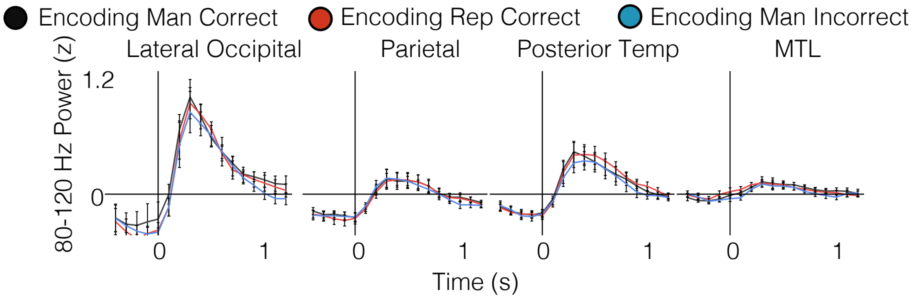

Figure S8 No differences in 80-120 Hz power in visual association cortex and MTL during encoding. Time courses of 80-120 Hz power across participants for LOC, PAR, PT, and MTL for the manipulated correct, manipulated incorrect, and repeated correct conditions locked to image presentation during encoding (200 ms sliding windows, 50% overlap; image appears at $t = 0$). No significant differences were observed for the manipulated correct compared to manipulated incorrect and repeated correct images ($p > .05$, permutation procedure). All error bars indicate standard error of mean.

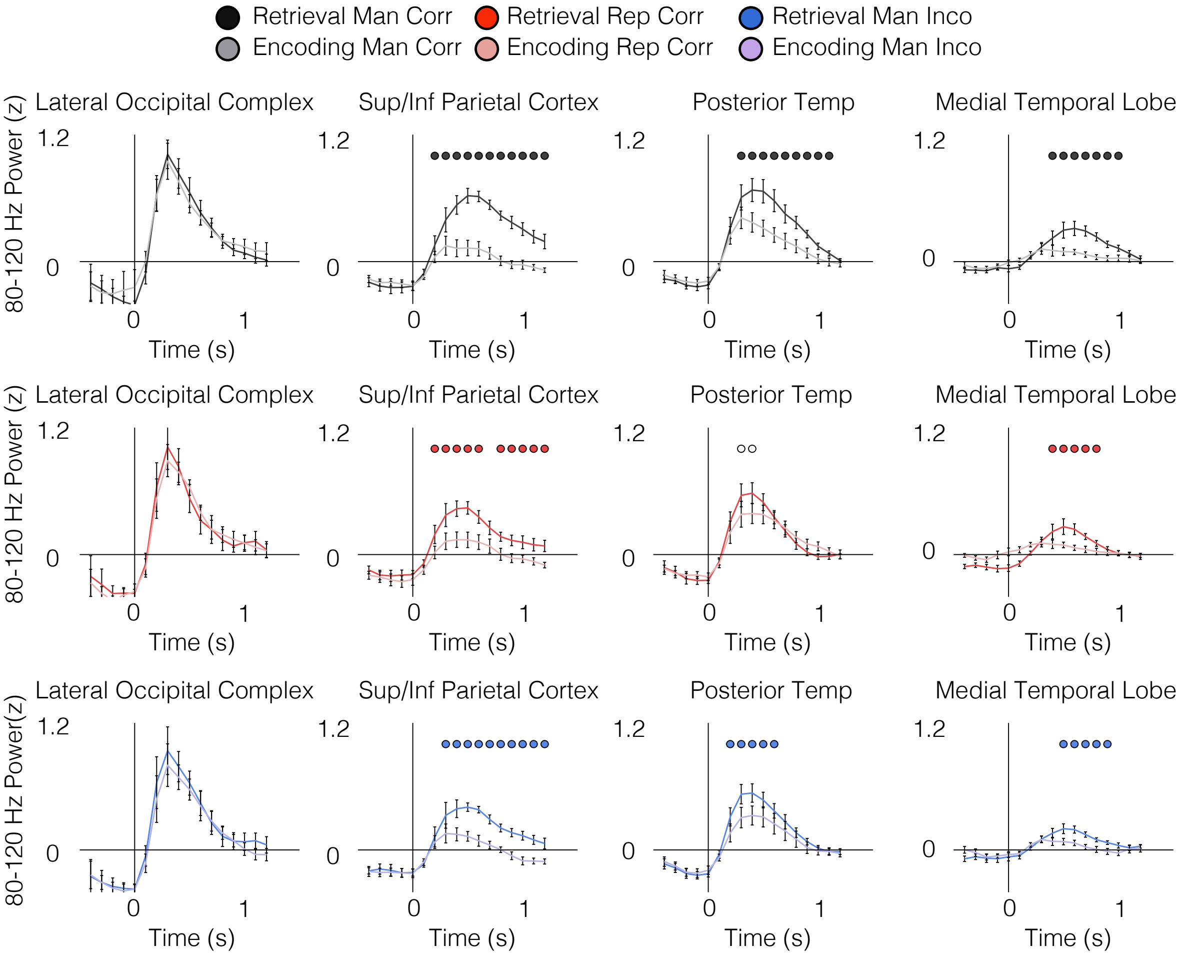

Figure S9 Increases in 80-120 Hz power in visual association cortex and MTL when comparing encoding and retrieval periods. Time courses of 80-120 Hz power for 6 LOC, 8 PAR, 10 PT, and 10 MTL participants for the manipulated correct, manipulated incorrect, and repeated correct conditions during encoding and recognition (200 ms sliding windows, 50% overlap; image appears at $t = 0$). Blue, red, and black dots indicate significant increases in power during the recognition period compared to encoding ($p < 0.05$, permutation procedure). White dots indicate significant increases in power that did not survive correction for multiple comparisons. All error bars indicate standard error of mean.

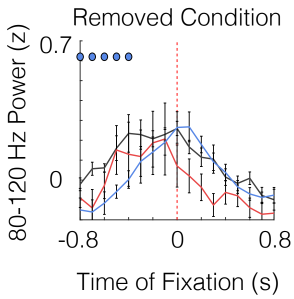

Figure S10 Increases in 80-120 Hz power locked to eye fixations on removed objects. Time courses of 80-120 Hz power for visually responsive electrodes across participants for the removed correct, removed incorrect, and repeated correct conditions locked to the first fixation on the region of the removed item (200 ms sliding windows, 50% overlap; eye fixation at $t = 0$). Blue and red dots indicate significant differences in 80-120 Hz power between removed correct and removed incorrect and repeated correct condition across 8 participants, respectively ($p < .05$, permutation procedure). All error bars indicate standard error of mean.

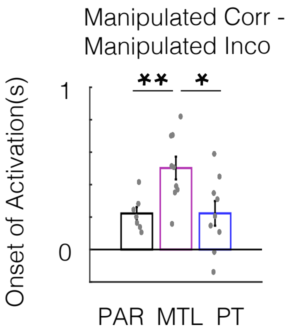

Figure S11 Differences in 80-120 Hz power between present and remembered visual experience emerge in visual association cortex earlier than in the MTL. Average estimated onset of differences in 80-120 Hz power between manipulated correct and incorrect conditions conditions in 9 PT, 7 PAR, and 9 MTL participants. Asterisks (*,**) indicate significance at $p < .05$ and $p < .01$, two-sided unpaired t-test. The differences in 80-120 Hz power between manipulated correct and manipulated incorrect conditions arose significantly earlier in PT and PAR compared to MTL (PT v MTL, $t(16) = -2.70$, $p = .016$; PAR v MTL, $t(14) = -3.21$, $p = .006$). All error bars indicate standard error of mean.

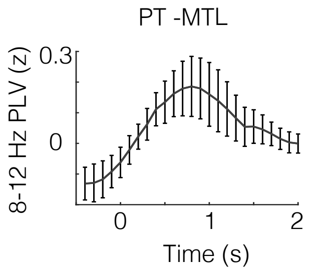

Figure S12 Increases in PT and MTL 8-12 synchrony after image presentation. Average 8-12 Hz phase-locking value (PLV) between PT and MTL electrodes locked to image presentation across 8 participants during recognition (200 ms sliding windows, 50% overlap; image appears at $t = 0$). All error bars indicate standard error of mean.

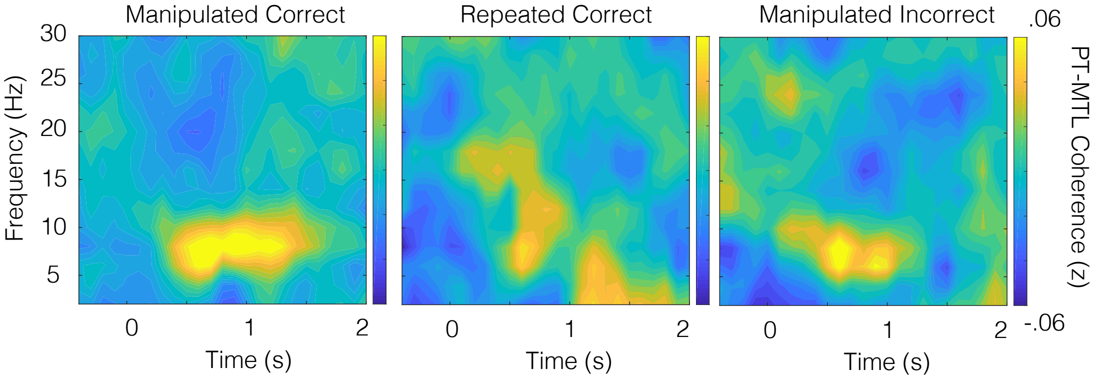

Figure S13 Average PT-MTL coherence spectrum for each condition. Average coherence spectrum for all PT-MTL electrode pairs across participants for the manipulated correct, manipulated incorrect, and repeated correct conditions.

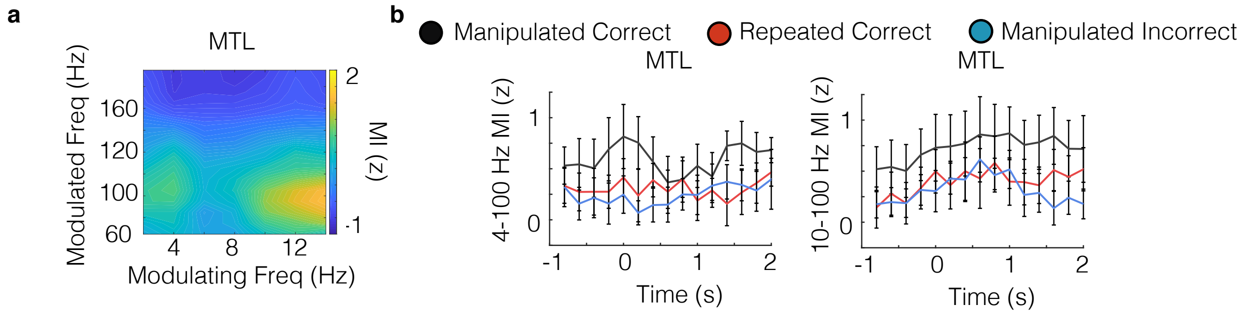

Figure S14 Phase amplitude coupling within the MTL across conditions **a)** Average normalized modulation index (MI) for all trial across all 10 MTL participants **b)** Average normalized MI index relative to image presentation (t=0) between 4 Hz and 100 Hz and between 10 Hz and 100 Hz for the manipulated correct (red), manipulated incorrect (black), and repeated correct (blue) conditions across all 10 MTL participants. **c)** Average normalized MI index between 10 Hz and 100 Hz across all 10 MTL participants. All error bars indicate standard error of mean.

1. Vaz, A. P., Inati, S. K., Brunel, N. & Zaghloul, K. A. Coupled ripple oscillations between the medial temporal lobe and neocortex retrieve human memory. *Science* **363**, 975–978 (2019).

2. Onslow, A., Bogacz, R. & Jones, M. Quantifying phase–amplitude coupling in neuronal network oscillations. *Progress in biophysics and molecular biology* **105**, 49–57 (2011).
