# Supplementary material for: Feedforward prediction error signals during episodic memory retrieval
Source: Nat Commun. 2020 Nov 27;11:6075. doi: 10.1038/s41467-020-19828-0 (PMC7699639; doi:10.1038/s41467-020-19828-0)
Supplement: Supplementary file 2 — Reporting Summary [file 41467_2020_19828_MOESM2_ESM.pdf]

nature research

Corresponding author(s): Kareem Zaghloul

Last updated by author(s): Oct 19, 2020

Reporting Summary

Nature Research wishes to improve the reproducibility of the work that we publish. This form provides structure for consistency and transparency in reporting. For further information on Nature Research policies, see our Editorial Policies and the Editorial Policy Checklist.

Statistics

For all statistical analyses, confirm that the following items are present in the figure legend, table legend, main text, or Methods section.

n/a	Confirmed	
☐	☒	The exact sample size (*n*) for each experimental group/condition, given as a discrete number and unit of measurement
☐	☒	A statement on whether measurements were taken from distinct samples or whether the same sample was measured repeatedly
☐	☒	The statistical test(s) used AND whether they are one- or two-sided<br>*Only common tests should be described solely by name; describe more complex techniques in the Methods section.*
☐	☒	A description of all covariates tested
☐	☒	A description of any assumptions or corrections, such as tests of normality and adjustment for multiple comparisons
☐	☒	A full description of the statistical parameters including central tendency (e.g. means) or other basic estimates (e.g. regression coefficient) AND variation (e.g. standard deviation) or associated estimates of uncertainty (e.g. confidence intervals)
☐	☒	For null hypothesis testing, the test statistic (e.g. *F*, *t*, *r*) with confidence intervals, effect sizes, degrees of freedom and *P* value noted<br>*Give P values as exact values whenever suitable.*
☒	☐	For Bayesian analysis, information on the choice of priors and Markov chain Monte Carlo settings
☒	☐	For hierarchical and complex designs, identification of the appropriate level for tests and full reporting of outcomes
☒	☐	Estimates of effect sizes (e.g. Cohen's *d*, Pearson's *r*), indicating how they were calculated

Our web collection on statistics for biologists contains articles on many of the points above.

Software and code

Policy information about availability of computer code

Data collection	Data were collected using the Nihon Kohden intracranial EEG (iEEG) acquisition system as well as using the Blackrock Microsystems LLC Cerebus iEEG acquisition system.
Data analysis	All analyses were performed using custom built Matlab 2018 (Natick, MA) code. FSL Brain Extraction Tool v6.0, OsiriX 11.0. FLIRT software contained within FSL packages. All custom code are available upon request.

For manuscripts utilizing custom algorithms or software that are central to the research but not yet described in published literature, software must be made available to editors and reviewers. We strongly encourage code deposition in a community repository (e.g. GitHub). See the Nature Research guidelines for submitting code & software for further information.

Data

Policy information about availability of data

All manuscripts must include a data availability statement. This statement should provide the following information, where applicable:

- Accession codes, unique identifiers, or web links for publicly available datasets
- A list of figures that have associated raw data
- A description of any restrictions on data availability

Processed data used in this study can be found at: https://dir.ninds.nih.gov/ninds/zaghloul/downloads.html

Field-specific reporting

Please select the one below that is the best fit for your research. If you are not sure, read the appropriate sections before making your selection.

[x] Life sciences [ ] Behavioural & social sciences [ ] Ecological, evolutionary & environmental sciences

For a reference copy of the document with all sections, see nature.com/documents/nr-reporting-summary-flat.pdf

Life sciences study design

All studies must disclose on these points even when the disclosure is negative.

Sample size	Due to clinical considerations, each participant has a different arrangement of implanted surface and depth electrodes. Given this variability and based on our previous work, we estimated that we need a minimum of 5 participants to perform a task before we can draw significant conclusions regarding changes in neural activity in one brain region. We estimated this minimum number of 5 since, using a standard permutation test in which each subject is the unit of observation and in which the values of each observation are permuted, one would need a minimum of 5 different observations (subjects) to find an effect with a significant p-value (< .05). The data in this study are drawn from a sample size that exceeds this minimum threshold.
Data exclusions	We implemented a previously reported automated trial and artifact rejection procedure base on excessive kurtosis or variance in iEEG signals. We also removed trials with response times greater than 10 seconds with the assumption that these trials were periods when the participant was not attending to the stimulus. These exclusion criteria were pre-established and consistent with our previous studies.
Replication	We did not replicate these results in a separate cohort of participants. The data presented here were captured over two years from intracranial recordings in human neurosurgical patients receiving treatment for epilepsy, and are thus extremely rare.
Randomization	Randomization of participants was not relevant to this study and participants were therefore not assigned to separate groups. Each participant completed experimental sessions in which analyses were conducted comparing neural activity between task conditions.
Blinding	Blinding was not relevant to this study. All participants performed the same behavioral task, and all analyses were performed by comparing the performance of each individual participant within one task condition versus another.

Reporting for specific materials, systems and methods

We require information from authors about some types of materials, experimental systems and methods used in many studies. Here, indicate whether each material, system or method listed is relevant to your study. If you are not sure if a list item applies to your research, read the appropriate section before selecting a response.

Materials & experimental systems

n/a	Involved in the study	
x		Antibodies
x		Eukaryotic cell lines
x		Palaeontology and archaeology
x		Animals and other organisms
	x	Human research participants
x		Clinical data
x		Dual use research of concern

Methods

n/a	Involved in the study	
x		ChIP-seq
x		Flow cytometry
x		MRI-based neuroimaging

Human research participants

Policy information about studies involving human research participants

Population characteristics	14 participants (7 male, 40.9 +/- 12 years) with intracranial electrodes placed for seizure monitoring.
Recruitment	Participants were recruited on the basis of clinical need for surgical epilepsy localization. All patients receiving intracranial electrodes were eligible to participate in this study, and there were no patients that were selected or excluded based on patient characteristics.
Ethics oversight	The Institutional Review Board of the National Institutes of Health and the National Institute of Neurological Disorders and Stroke approved the experimental protocol (11-N-0051 Epilepsy Surgery) through which these data were collected.

Note that full information on the approval of the study protocol must also be provided in the manuscript.
